# Livestock husbandry in Islamic Cártama, Málaga, Spain: the micro and bioarchaeology of an open-air *Fumier* sequence

**DOI:** 10.1007/s12520-025-02345-w

**Published:** 2025-11-20

**Authors:** Rowena Y. Banerjea, Mónica Alonso Eguiluz, Lionello F. Morandi, Jérôme Ros, Luc Vrydaghs, Yannick Devos, Marie Larrieu, Nicolás Losilla, Irene Bertelli, Erika Ribechini, Ana Medina Cuesta, Marcos García García, Francisco Melero García, Guillermo García Contreras, Aleks Pluskowski

**Affiliations:** 1 Department of Archaeology, School of Archaeological, Geography and Environmental Science, University of Reading, Whiteknights, Reading, UK; 2 Archaeology, Environmental Changes and Geo-Chemistry (AMGC), Vrije Universiteit Brussels, Brussels, Belgium; 3 Dipartimento di Civiltà e Forme del Sapere, Università di Pisa, Pisa, Italy; 4 Institut des Sciences de l’Evolution , Université Montpellier, CNRS, IRD, EPHE, UMR5554, Montpellier, France; 5 Departamento de Historia Medieval y Ciencias y Técnicas Historiográficas, Universidad de Granada, Granada, Spain; 6 Dipartimento di Chimica e Chimica Industriale, Università di Pisa, Pisa, Italy; 7 Dipartimento di Scienze dell’Antichità, Università degli Studi di Roma “La Sapienza”, Rome, Italy; 8 ARATISPI Patrimonio S.L, Antequera, Spain

## Abstract

Multidisciplinary micro- and bioarchaeological analyses of late medieval deposits from suburban Cártama, Málaga, Spain, have, for the first time, confidently identified open air *fumier* deposits, which are supported by preliminary analysis of the faunal assemblage. This demonstrates that Andalusi livestock husbandry practices included periodically burning animal dung within corrals and pens, which is a long-standing practice in the Mediterranean region also observed in prehistoric transhumance. These techniques have commonly been applied to prehistoric *fumier* or pastoral sites and now demonstrate the importance of expanding micro- and bioarchaeological work to *fumier* sequences from other periods. This research from Cártama offers a window into the so-called medieval “Green Revolution” associated with Arab migrations and highlights the necessity of identifying in situ penning deposits on archaeological sites and of applying high resolution analyses to examine them. The analyses reveal data on animal feeds, including pearl millet (*Pennisetum glaucum*) and sorghum (*Sorghum bicolor*) which are rare in the Iberian Peninsula at this time, the use of plant resources in livestock husbandry, and the preparation of fertiliser, which are central to understanding farming practices in Al-Andalus.

**Supplementary Information:**

The online version contains supplementary material available at 10.1007/s12520-025-02345-w.

## Introduction

**Fig. 1:**
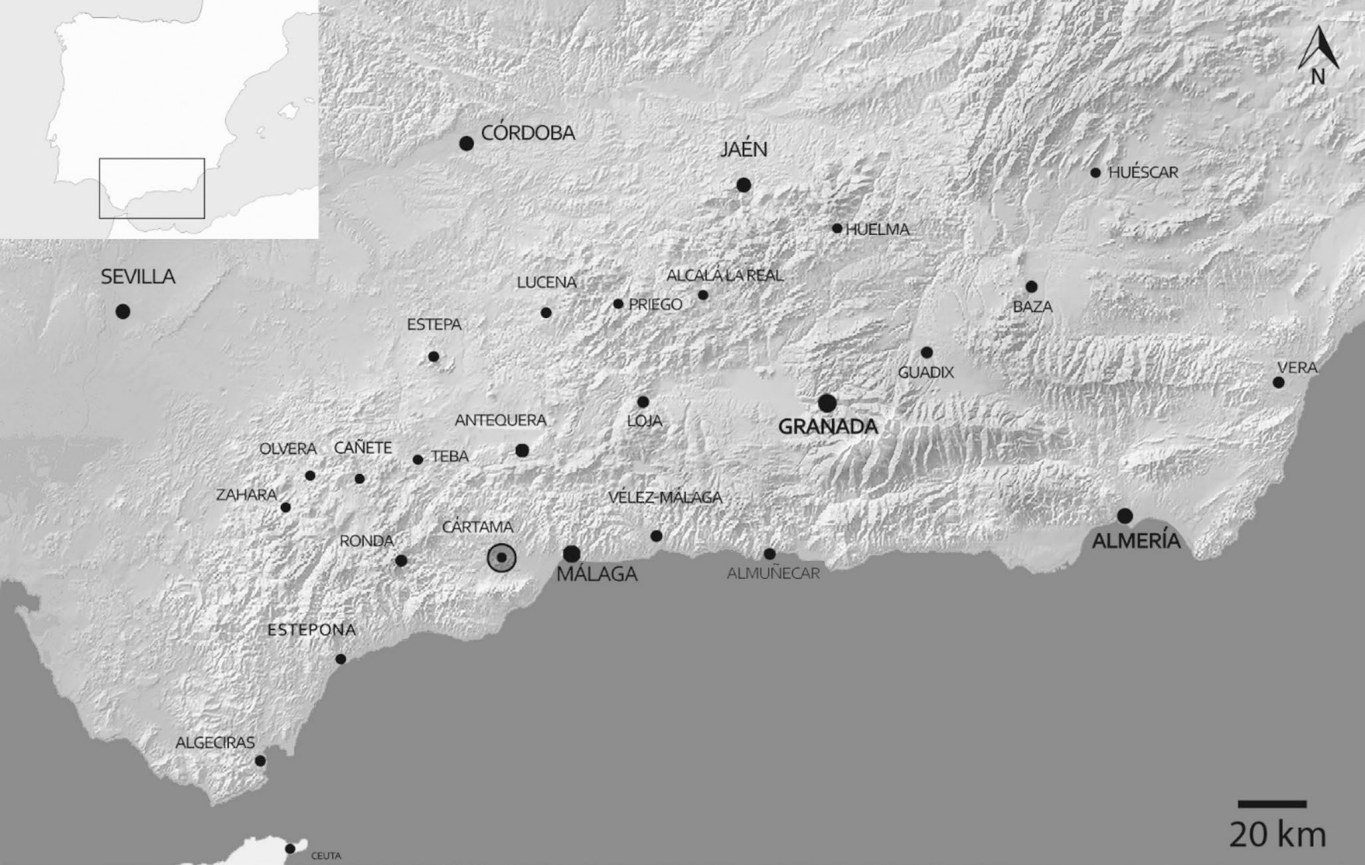
Map showing the location of Cártama in present-day Andalusia, Spain

Knowledge regarding livestock husbandry in Al-Andalus is derived from contemporary written sources, in which it is scarcely mentioned (García-García and Moreno-García 2018), ethnohistory (e.g. Jiménez Castillo et al. 2023) and a developing body of zooarchaeological research (e.g. García-García 2017, 2023; Grau-Sologestoa 2017) and isotopic studies (e.g. Alexander et al. 2019; Inskip et al. 2019). The livestock of the former kingdom of Granada is the best known in Al-Andalus due to the relative abundance of written documentation generated during the Nasrid period (1232–1492) and the period immediately following the Castilian conquest (1492-). One of the main conclusions from the study of the available written sources, which are mainly devoted to the analysis of agrarian systems, is the comparatively low importance of livestock farming during this period. Pastoralism is an exceptionally opaque reality in written documentation and rarely appears in these sources (García-García and Moreno-García 2018). This paper considers archaeological evidence from a late medieval suburban context in Cártama (Málaga province, Fig. 1) which sheds new light on husbandry practices in Al-Andalus. This, in turn, offers a window onto the so-called medieval “Green Revolution” associated with Arab (and in Iberia, also Berber) migrations.

This research from Cártama makes two significant contributions:

1. To archaeological science as the first published example of an open-air archaeological *fumier* sequence.
2. To knowledge about the livestock sector in Al-Andalus, which is under-studied in comparison to irrigated agriculture and scantly represented in documentary sources.

The archaeological deposits that are analysed in this research relate to the period when Cártama, in Al-Andalus, was under Muslim rule as part of the Almohad Caliphate in the “Islamic West”. The concept of an Islamic “Green Revolution”’ was popularised by the economic historian Watson (1974). It refers to the introduction of fundamentally new agrarian practices within regions that came under Muslim rule in south-western Asia and the Mediterranean. Watson argued that crop diffusion of 18 new species (including rice, sorghum, bananas, watermelon and cotton) alongside novel agricultural technologies (especially irrigation and summer cropping), was facilitated by Arab migrations, leading to an intensification in arable production. This, in turn, was a catalyst for economic and societal changes that has left an agrarian legacy which also impacted neighbouring societies – in the case of the Mediterranean, this included Christian Europe. Recent developments in archaeobotany, scientific dating and landscape archaeology have critiqued the tempo and character of Watson’s proposed “Green Revolution” (Watson 1974), particularly in relation to the Iberian Peninsula. This model can be reframed by connecting production with consumption (Kirchner et al. 2023) and, of particular relevance to Cártama, the links between cultivation and the livestock sector. Kirchner et al. (2023) argue for the application of, alongside data on land use, organic residue analysis, sedaDNA, palynology and stable isotope analyses of animal bones to trace the intensity and spatial distribution of large-scale livestock rearing and foddering practices. The application of soil micromorphology provides a key and fundamental tool for identifying and interpreting in situ stabling deposits on archaeological settlements (eg. Banerjea et al. 2020; Banerjea et al. 2021; García-Suárez et al. 2018; Polo Díaz and Fernández Eraso, 2010; Polisca et al. 2025) and is applied to a series of deposits in this study from an archaeological feature at Cártama, from which plant macroremains, phytoliths, pollen, non-pollen palynomorphs (NPPs), and faecal lipid markers are studied in-tandem.

### The archaeological context

Cártama, Málaga, is an urban centre in the south of Spain (Fig. 1) with a long history from the 8th century BCE through to the present-day town. This historic centre has its origins at least from the last stage of the Late Bronze Age-Ancient Iron Age with a protohistoric settlement followed by an Iberian city, the Roman Cartima and another Byzantine-Visigothic enclave prior to the Islamic occupation.

Cártama is one of the geographical areas in Málaga with the best primary sources for the early medieval period (al-Jusani or Ibn al-Faradi). Yemeni Arabs occupied the most fertile and urbanized agricultural areas in the south of the Peninsula, in this case the Guadalhorce Valley. Colonization took advantage of existing urbanized spaces -*civitas*- and their network of dependent farmhouses (villages). The building of the fortress reflects trends typical of both the Almohad and Nasrid eras, especially the latter. A *maqbara* (Muslim graves of religious figures) associated with the fortress and an existing cemetery on the other side of the Los Chorritos stream were identified through excavation. The analyses here pertain to an area of medieval dumps outside the fortress that were used throughout the Andalusi period and related to the houses in the suburbs that the texts refer to.

**Fig. 2:**
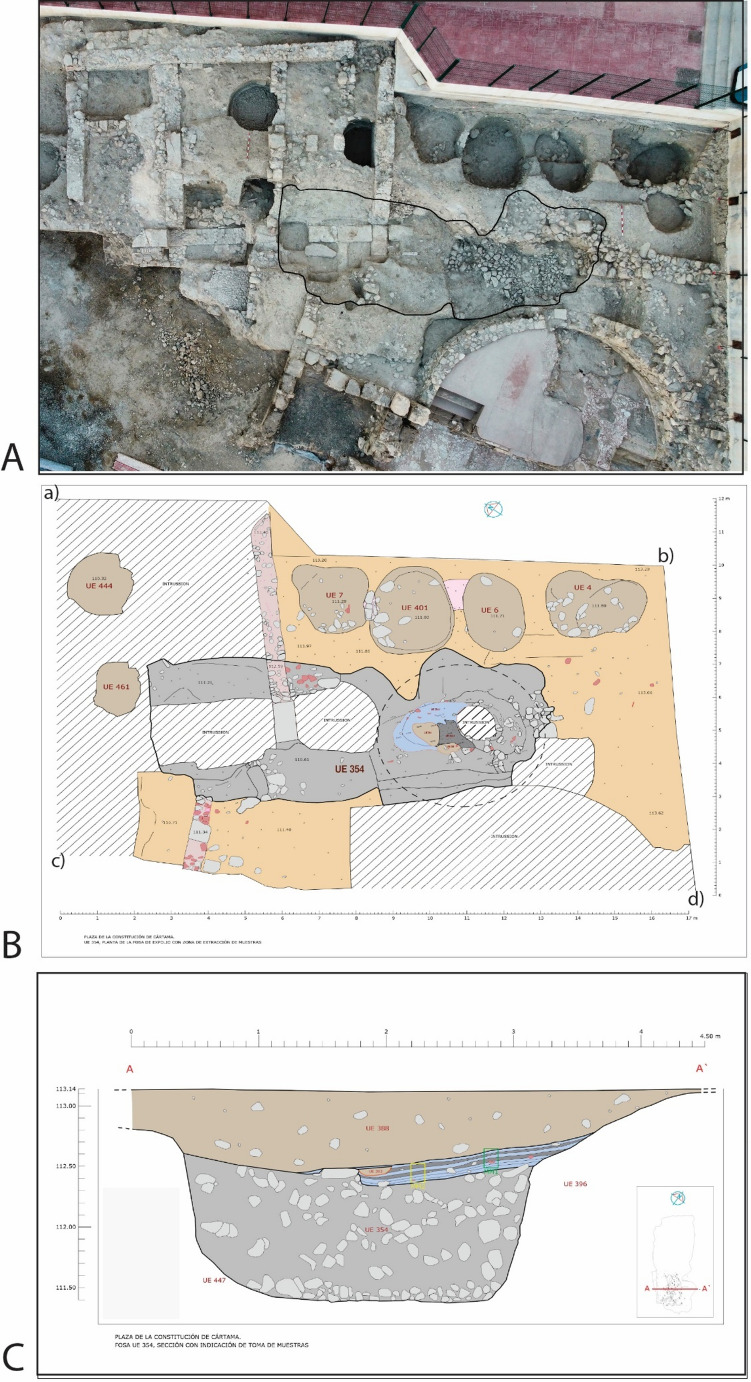
Showing the alignment of UE 354 (**A**); the maximum extension of UE 354 and later intrusion with geoposition locations (a) X 354326.19, Y 4063951.09, (b) X 354334.19, Y 4063936.69, (c) X 3543263.16, Y 4063931.07, (d) X 354326.78, Y 4063930.19 (**B**); and the profile through UE 354. The depth of the profile is recorded in metres above sea level (**C**)

**Fig. 3:**
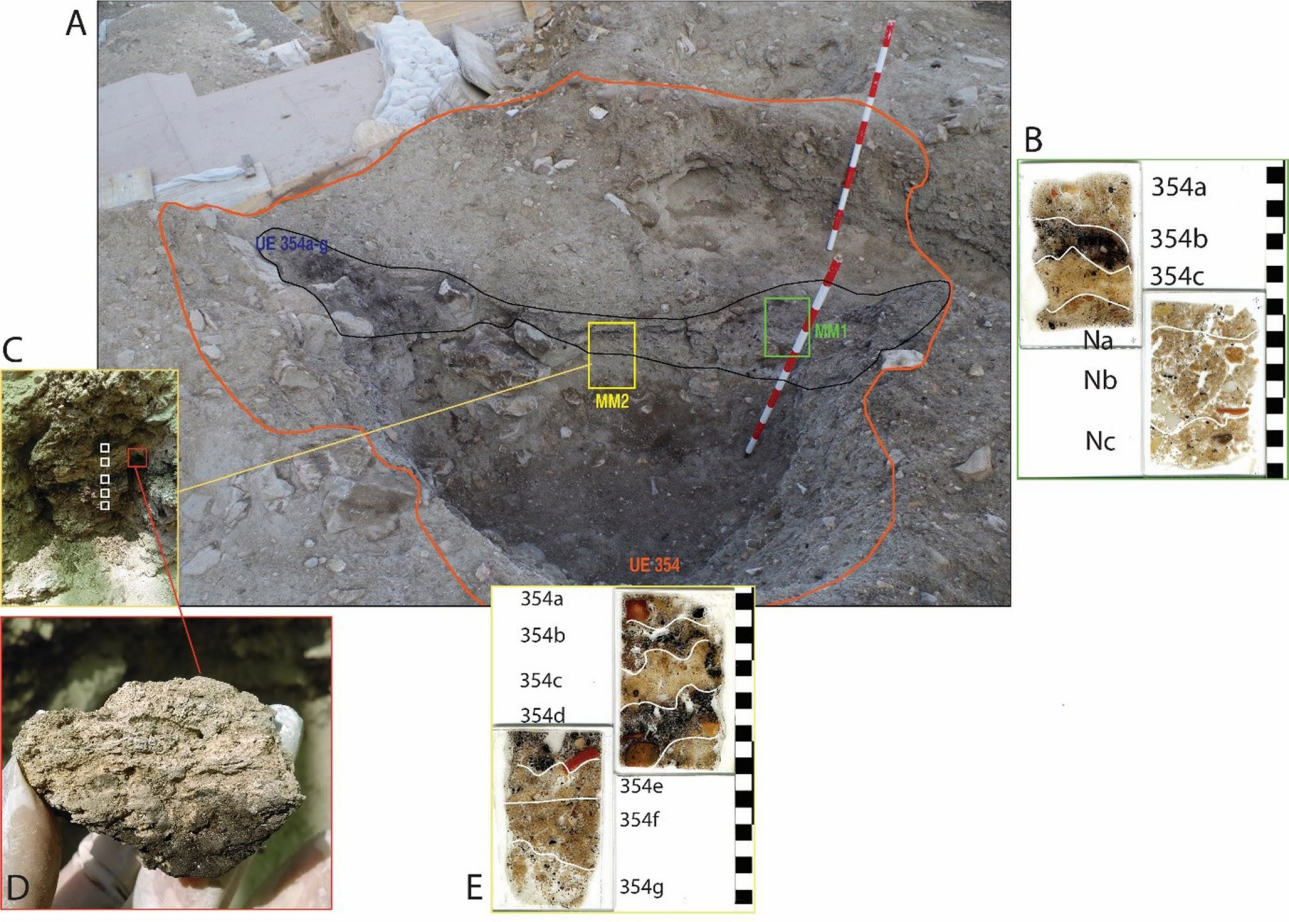
**A**: Photograph of the profile through UE 354. The irregular outline of UE 354 is marked in orange. Micromorphology samples MM1 and MM2 are marked in green and yellow respectively. **B**: Microstratigraphic Units within MM1; **C**: Alternating blackened and un-blackened sediments in MM2 and the locations of spot phytolith samples 1–5 (top to base) collected in 2019 which are marked by white boxes. **D**: Plant impressions that are visible to the naked eye as voids or white striations throughout an un-blacked layer, MU 354c. **E**: Microstratigraphic Units within MM2. Microstratigraphic Units 354a-c were excavated in 2021 and assigned UE 354.2–354.4.4 (see Table 1)

UE 354 is recorded in the southeast area of the site, where the stratigraphy of this period is best preserved. The alignment of UE 354 coincided with that of a Roman wall that was looted (Fig. 2A, B), which shows its use as a looting pit, measuring 10.80×4.30 m and 1.60 m deep. UE 354 was filled with different strata (Fig. 2C): first rubble and later terrigenous sediments, among which was a thick layer of ash and incinerated materials (later identified as UE 354a−f following soil micromorphological analysis) (Fig. 3A), with a maximum extension of 4.00×3.40 m and a thickness of 0.20 m, which was partially affected by a later intrusion (Fig. 2B). The abundant ceramic materials recovered from UE 354 give us a late Almohad chronology, which we can clearly establish in the early part of the 13th century. It is a time when *ataifores* (bowl or deep plate characteristic of Al-Andalus ceramic production) glazed in copper oxide green clearly predominate over molasses, and where sgraffito jugs, casseroles and pots are found. A small faunal assemblage was recovered and is being analysed.

Radiocarbon dates on four carbonised seeds and one sample of (poorly preserved) bone collagen were determined by Beta Analytic. The seeds show a late Almohad to early Nasrid transition for the sequence, with most calibrated dates falling within mid−12th to mid−13th century CE. The sample of bone collagen produced a prehistoric date ranging from 765 − 464 cal BCE (93.3%) and the taphonomic factors relating to this are discussed below.

### Rationale for a “*fumier*” sequence

**Fig. 4:**
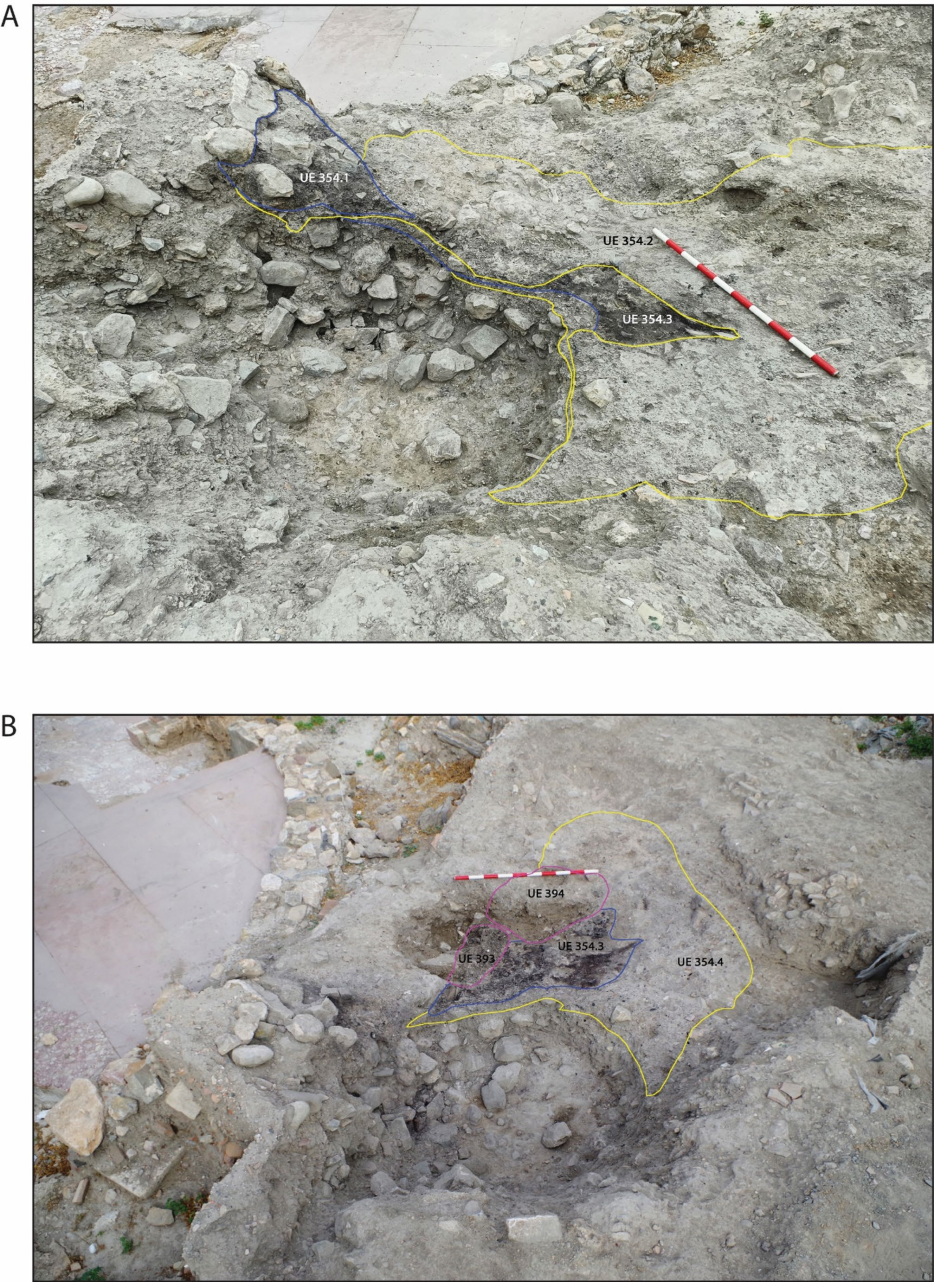
(**A**) UE 354.1, UE 354.2 and UE 354.3 defined in excavation; (**B**) *Fumier* layer UE 354.4 and pits UE 393 and UE 394 defined in excavation

The alternating blackened and un−blackened sediments (Fig. 3 C; Fig. 4 A) in the profile were particularly distinctive during excavation and plant impressions were visible to the naked eye in UE 354.4, pale, un−blackened sediment (Fig. 3D). Soil micromorphological analysis identified that UE 354 comprised a series of microstratigraphic units (Fig. 3B & E), which were excavated in 2023 and, through further bio−and micro archaeological analyses, it is argued here that this feature is not a pit, but a corral represented by a *fumier *sequence. Two pits were dug into the *fumier *sequence, UEs 393 and 394 (Fig. 4B), the fills of which were also sampled for macrobotanical and phytolith analysis.

*Fumier* is a French word that translates to “dung” or “manure”. It refers to animal waste, such as excrement from stables and barnyards. In archaeology, *fumier* sequences play a primary role in the study of livestock management and the use of space in prehistoric pastoralist societies. They are formed when dung accumulated in sheepfold caves and rock shelters is burnt with other plant remains, resulting in an overlapping of burnt and unburnt sedimentary layers. The scientific study of these sedimentary layers (deposits) informs us about livestock diet and the use of vegetal resources in animal husbandry (Alonso-Eguiluz et al. 2017, 2024; Angelucci et al. 2009; Brochier et al. 1992; Delhon et al. 2024; Macphail et al. 1997; Morandi 2025; 2020; Polo Díaz and Fernández Eraso, 2010; Polo-Díaz et al. 2016). According to the literature (Angelucci et al. 2009) on *fumier* sequences, only one sequence is considered to have been identified outside of the usual rock shelter setting, in the open air, and is not published.

The key field and bio- and microarchaeological observations of a *fumier* sequence are:

- Alternating black and white sub-horizontal layers in the stratigraphic sequence, clearly observable in profile, very difficult to excavate in plan due to variability in the burning across the floor. The whitish layer is normally related to the plant material (maybe the remains of the wood corral’s fence) that has been set alight to burn the dung.
- The scarce artefacts that do appear are heavily altered by high temperature combustion.
- Presence of dung.
- Presence of bedding.

The first two of the above bullet points were observable in the field (Figs. 3 and 4). The presence of dung, bedding and other plant materials and evidence for burning are all reported here as a result of multi-proxy bio- and micro-archaeological analyses. Soil micromorphology, macrobotanical, phytolith and organic residue analyses have been routinely applied to the examination of deposits from *fumier* sequences and pastoral sites (Alonso-Eguiluz et al. 2024; Angelucci et al. 2009; Macphail et al.^1997^; Polo Díaz and Fernández Eraso, 2010; Polo Díaz et al. 2016; Vallejo et al. 2022). Palynology, and specifically the analysis of non-pollen palynomorphs such as spores of coprophilous fungi, is less routinely applied anthropogenic deposits (Delhon et al. 2024), perhaps except for those that are waterlogged (e.g. Banerjea et al. 2020; 2021; Maslin 2018). However, non-pollen palynomorphs were fundamental to identifying a non-waterlogged, medieval animal enclosure at Beckey Chapel, UK (Banerjea et al. 2021) and, also with pollen, can provide good records of human practices (Delhon et al. 2024 and references therein).

## Materials and analytical methods

### Sampling and excavation

**Fig. 5:**
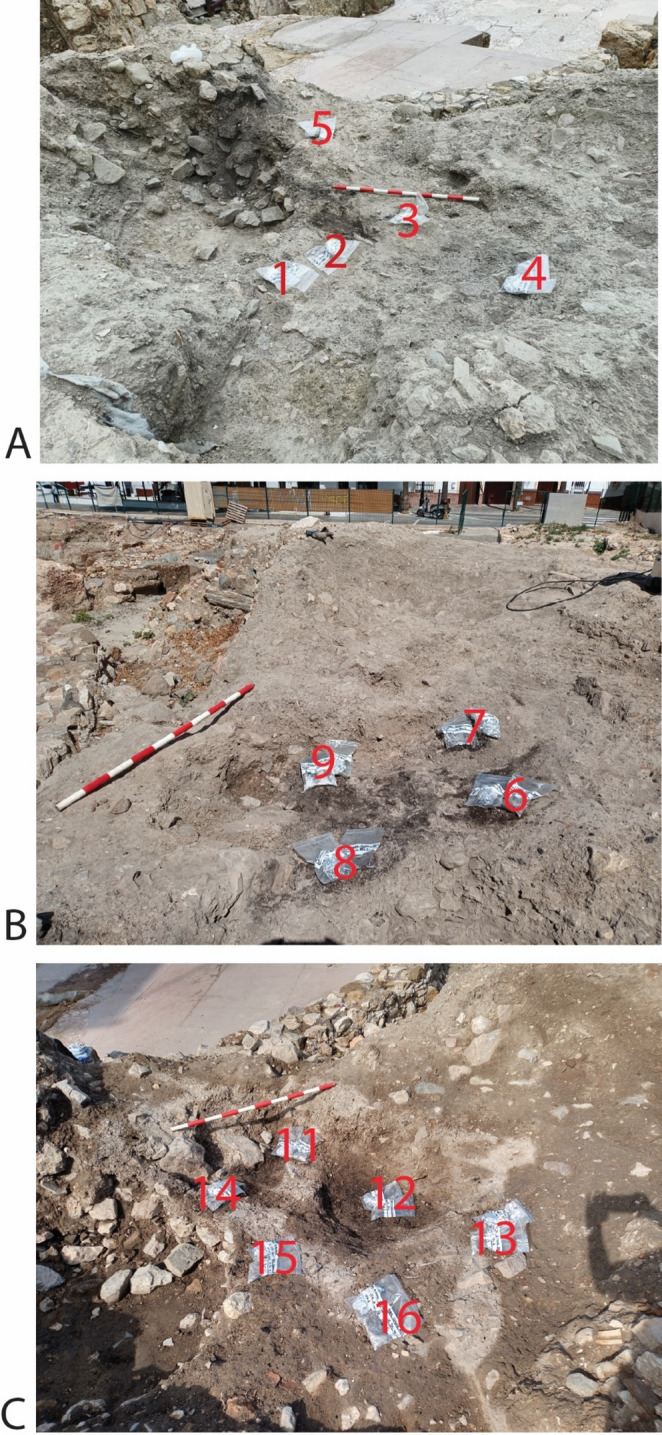
Showing the numbered locations of samples, which correspond to sample numbers in Table 1, in: **A**: UE 354.2; **B**: UE 354.3; and **C**: UE 354.4

Excavation started in 2016, with two phases of sampling: the first in 2019; the second in 2023. In 2019, two samples for soil micromorphological analysis and spot samples for phytolith extractions were collected from a profile that had already been exposed by the excavation of an intercutting pit into the feature UE 354 (Fig. 3). Desiccated plant remains, and plant impressions (from decayed plants) were observable during excavation (Fig. 3D). The micromorphological analysis and a phytolith assessment were conducted after the initial fieldwork in 2019 and the results informed the small excavation in 2023 to spatially collect samples for phytolith analysis, palynology, characterisation of faecal lipid markers and plant macroremains. In 2023, several of the burnt and unburnt layers were assigned separate context (UE) numbers, which were informed by soil micromorphological analysis and the identification of microstrigraphic units (MU) of the samples collected in 2019, and were defined, recorded, then sampled and removed (Figs. 4 and 5; Table 1). Not all MU were excavated in 2023 due to time constraints. The excavation was a challenging process due to the intercalation of the layers and lateral discontinuation, which is a feature of *fumier* sequences caused by the irregular burning pattern across the surface (Macphail et al. 1997; Angelucci et al. 2009).

**Table 1:** Showing stratigraphic units (UE) assigned in 2023, microstratigraphic units defined in 2019, associated samples and their location of collection (as marked on Figs. 3 and 5).N/S = Not sampled. N/A = Not applicable

Feature	UE from 2023	Plant Macros sample (# bags)	Location # on photo (Fig. 5)	Biomarkers (BIO) sample number	Phytolith & Palynology sample number	2019 Phytolith spot from profile (Fig. 3C)	Related microstratigraphic unit (MU) (Fig. 3)
Fumier	354.2	20 (6)	1	8	13	N/A	354a
			2	9	14		
			3	10	16		
			4	11	17		
			5	12	18		
	354.3	35 (10)	6	27	31	1	354b
			7	28	32		
			8	29	33		
			9	30	34		
	354.4	55 (8)	11	43	49	2	354c
			12	44	50		
			13	45	51		
			14	46	52		
			15	47	53		
			16	48	54		
	Not excavated	N/S	N/A	N/S	N/S	3	354d
		N/S	N/A	N/S	N/S	4	354e
		N/S	N/A	N/S	N/S	5	354f
Pit	354.1.1	6 (1)	N/A	N/S	N/S	N/A	N/A
Pit	393	23 (1)	10	25	24	N/A	N/A
Pit	394	(3)	N/A	N/S	N/S	N/A	N/A

### Archaeological soil and sediment micromorphology

Four thin-sections, 11.5 × 7.5 cm in size, were prepared in the Microanalysis Preparation Unit, University of Reading, from two blocks (two thin-sections per block) that were collected from the profile through UE 354 (Fig. 3). The procedure followed the University of Reading standard protocol for thin-section preparation (see Banerjea et al. 2021).

Micromorphological investigation was carried out using a Leica DMLP polarising microscope at magnifications of 40x – 630x under Plane Polarised Light (PPL), Crossed Polarised Light (XPL), and where appropriate Oblique Incident Light (OIL). Thin-section description was conducted using the identification and quantification criteria set out by Bullock et al. (1985) and Stoops (2003) with reference to Mackenzie and Adams (1994) and Mackenzie and Guilford (1980) for rock and mineral identification, Nicosia and Stoops (2017) for the identification of inclusions, and Stoops et al. (2018) for further identification of post-depositional features and processes. Phytoliths were described according to Vrydaghs and Devos (2018) and Kaczorek et al. (2018). Photomicrographs were taken using a Leica camera attached to the Leica DMLP microscope and using a ZEISS petrographic microscope with a Deltapix camera.

### Carpological and anthracological remains

A total of 6 samples was studied (UE 354.2, 354.4, 393, 394, plus 2 samples with no specific context indication) whose initial volume ranged from 1.8 to 18 L of raw sediment. The protocol for extracting the archaeobotanical remains consisted of sieving the sediments by flotation on 2 mm and 250 μm meshes, using a home-made flotation machine operated by a domestic electric pump. The extraction and identifications of the archaeological seeds were carried out with the aid of a Nikon SMZ645 stereoscopic magnifying glass, with magnifications from 6.5x to 10x. As an anatomical comparison was required for the observations and identifications of the carpological remains, we used the “Institute of Evolutionary Science of Montpellier (ISEM)” reference collection of fresh and archaeological seeds, as well as the following publications: Jauzein 1995, Cappers et al. 2006; Jacomet 2006, Sabato and Peña-Chocarro 2021. The observation of anthracological remains was carried out using a reflection microscope, Olympus BH2-UMA (100x, 200x and 500x). The identification was carried out using the reference collection of the ISEM bioindicator platform, as well as reference atlases (Schweingruber 1990; Vernet et al. (2001). Photographies of charcoal fragment of interest were taken using the Hitachi JEOL TEM/SEM from MEA platform (Université de Montpellier).

### Phytolith analysis

Sixteen sediments bulk samples (BS) were analysed. Phytolith extractions were carried out following Katz et al. (2010). Between 20 and 50 mg of sediment was placed in a 0.5 mL Eppendorf tube and 50 µL of HCl 6 N was added. After the reaction, 450 µL of sodium polytungstate solution (SPT) [Na6(H2W12O40)∙H2O] with a density of 2.4 g/mL was added. The tube was vortexed and sonicated for 5 min and then centrifuged for 5 min at 5000 rpm. The supernatant liquid was removed and transferred to another tube. 50 µL of the aliquot was placed on a microscope slide and covered with a 24 × 24 mm coverslip. BS were analysed at the Vrije Universiteit Brussels under a ZEISS petrographic microscope. Phytoliths present in 20 visual fields at 200x magnifications were counted for phytolith quantification, and morphological identification was carried out at 500x and 800x magnifications (if necessary).

Morphological identification of phytoliths was based on modern reference collection (www.phytcore.org) as well as standard literature (Piperno 2006; Twiss 1992). The nomenclature of the phytoliths followed the International Code for Phytolith Nomenclature 2.0 (ICPT, 2019), whenever possible. A minimum of 200 recognizable phytoliths were identified to obtain a reliable data set (Albert and Weiner 2001). Whenever possible, morphotypes were grouped into different categories based on their taxonomic and anatomical provenance: Poaceae ((C_3_ and C_4_), Poaceae leaves/stems, Poaceae inflorescences, and dicots. Due to their low taxonomic value, Elongate entire was not included in the analysis (ICPT, 2019). Phytoliths showing postdepositional alterations were classified according to three categories: fragments, resulting of mechanical processes; weathered phytoliths, suffering severe chemical alteration and the subsequent loss of attributions, making them unidentifiable; and melted phytoliths, presenting evidence of heating such as change in colour (darker colour) and bubbles on their surface. If thermal alterations are too extreme, phytoliths and other silica microremains lose their morphology completely appearing as vitrified silica (Alonso-Eguiluz et al. 2024). Observations were also made under UV (380 nm) and blue light (470 nm), in order to check if phytoliths were heated even though they show no melting signs (Devos et al. 2021; Vrydaghs et al. 2024).

### Palynology

Six grams of sediment were sieved through 125 μm and 10 μm meshes and treated with HCl to dissolve carbonates. Following deflocculation in Sodium pyrophosphate 1%, a known quantity of *Lycopodium* spores was added, and mineral matter was removed by means of heavy liquid separation (Sodium polytungstate, specific gravity 2.0 g/cm3). In order to avoid any potential damage to parasite eggs (Banerjea et al. 2021), a modified version of acetylation (Florenzano et al. 2012a) was carried out, heating the sample for a longer time (10 min) at a slightly lower temperature (90 °C). Untreated samples were also obtained to further check the presence of intestinal parasite eggs following heavy liquid separation. The residue was then diluted in liquid glycerol and examined in light microscopy at 400x and 600x magnifications.

### Faecal lipid marker characterisation

#### Analytical procedure

Each soil sample was powdered using a ball mill, to homogenise and increase the contact surface. 10 g of the obtained powder were subjected to alkaline hydrolysis by adding 10 mL of a hydroalcoholic solution of KOH (KOH at 10% in CH_3_OH: KOH at 10% in H_2_O/2:3), for 3 h in sonic bath at 60 °C. At the end of the hydrolysis, the supernatant was transferred into a conic vial and centrifugated at 3000 RPM for two minutes. The clear supernatant was acidified with HCl (solution 6 M) and then, neutral and acid organic components were extracted with hexane (300 µL for three times) and diethyl ether (300 µl for three times), respectively. The two fractions obtained were reunited and evaporated to dryness under nitrogen stream before being subjected to derivatization, which involved the use of 20 µL of N, O-bistrimethylsilyl-trifluoroacetamide (BSTFA) with 1% trimethylchlorosilane (derivatization agent), 150 µL of iso-octane and 5 µL of tridecanoic acid (internal standard of derivatization) solution at 60 °C for 30 min. Finally, 5 µL of hexadecane solution (internal standard of injection) were added to the solution before the injection (2 µL of these solution) in the GC/MS system.

#### Gas chromatography-Mass spectrometry (GC/MS)

Analysis was performed using a 6890 N GC system gas chromatograph (Agilent Technologies, Palo Alto, CA, USA) with a split/splitless injection, coupled with a 5975 mass selective detector (Agilent Technologies, Palo Alto, CA, USA) single-quadrupole mass spectrometer. The mass spectrometer was operated in the EI positive mode (70 eV) analyzing mass in the range m/z 50–650. For the chromatographic separation, an HP-5MS fused silica capillary column (5% diphenyl/95% dimethyl-polysiloxane, 30 m x 0.25 mm i.d., 0.25 μm film thickness, J&W Scientific Agilent Technologies, Palo Alto, CA) with a deactivated silica precolumn (2 m x 0.32 mm i.d., J&W Scientific Agilent Technologies, Palo Alto, CA) was used. The gas chromatographic conditions were as follows: initial temperature 80 °C, 2 min isothermal, then ramped at 10 °C/min up to 200 °C, 3 min isothermal, then ramped at 10 °C/min up to 300 °C, 45 min isothermal. The carrier gas was He (purity 99.9995%), at a constant flow rate of 1 mL/min. Peak assignment was based on the comparison with libraries of mass spectra (NIST 20 main EI MS library) and literature.

### Archaeofaunal remains

The analytical protocol follows García (2019), with most faunal remains manually recovered during excavation. While a sediment sample was taken for flotation, the resulting faunal remains have not yet been analysed. This may have introduced a bias in the assemblage against small-sized taxa and anatomical elements, although this limitation is not relevant for the results presented in this paper. All material was cleaned, sorted, and analysed at the University of Granada, and specimens were classified as either identified (NISP) or non-identified remains. The latter category includes undiagnostic fragments such as long bone shafts, vertebrae (except the atlas and axis), ribs, and non-diagnostic portions of scapulae (except the glenoid cavity and neck) and pelves (except the acetabulum). Taxonomic identification, particularly the distinction between sheep and goat, was only attempted on a limited set of anatomical elements. Criteria were drawn from various established sources, including Boessneck (1969) and Zeder and Lapham (2010) for postcranial bones, and Payne (1985) and Zeder and Pilaar (2010) for mandibular teeth. Age-at-death estimations were based on the state of epiphyseal fusion and dental eruption and wear stages, following O’Connor (1989) and Silver (1969) for bone fusion, and Payne (1973, 1987) for dental data. Anatomical representation was assessed through the calculation of MNE and MAU, derived from diagnostic zones and adjusted to expected skeletal frequencies. MAU values were subsequently normalised (MAU/E) to detect over- and underrepresented elements, following the protocol developed by O’Connor (2003: 144–147; see also García 2019). Butchery marks were systematically recorded using anatomical templates (Popkin 2005), noting the type of modification, its location on the bone, and the inferred purpose, in accordance with Binford (1981).

## Results

### Archaeological soil and sediment micromorphology

The results of the soil micromorphological analysis are presented in a summary table (Table 2) and the full dataset is available as supplementary data tables: sediment descriptions (Sup. Table 1); percentages of inclusions (Sup. Table 2); and post-depositional alterations (Sup. Table 3). The MUs were classified according to the description of their sediment attributes (such as particle size, sorting, the related distribution of the coarse and fine materials and the orientation and distribution patterns of the inclusions) and their inclusions. This identifies the origin of the materials, the depositional processes and preservation and post-depositional alterations. Classification reveals that the profile from Cártama comprises animal penning and discard deposits, some of which are in situ and some which are mixed and have been reworked by bioturbation. MUs Na, Nb and Nc were not able to be linked by a direct stratigraphic relation to the other MUs, including those excavated in 2023. Therefore, they are marked as “new”.

**Table 2:** Summary table of soil micromorphology results. Key: ●●●●● very abundant > 20%; ●●●● abundant 10–20%; ●●● many 5–10%; ●● occasional 2–5%; ● Rare < 2% (Bullock et al. 1985). Y = Yes

Deposit type	Sample	Context number	MU number	Inclusions					Sediment description			Microstructure effects						Decay		Excremental pedofeatures
				% geology	% building materials	% domestic refuse	% dung indicators	% organic	Key attributes	Colour	Strongly oriented lenses of organics	Lenticular platey peds	Crumbs or granules	SA Blocky peds	Cracks	Infillings	Mesofaunal / root bioturbation: channels and chambers	P nodules or staining	Organic staining	Earthworm granule
Reworked discard and penning deposits	Upper (MM1) A	354	354a	22.5	10	17.5	25	25	Sandy clay loam. Unsorted. Linked and coated/ Intergrain aggregate.	PPL: greyish brown; XPL: golden brown.	Y		Y							
Penning deposits in situ	Upper (MM1) A	354	354b	10	10	10	32.5	37.5	Sand/ loamy sand. Unsorted. Linked and coated.	PPL: greyish brown/ very dark brown; XPL: yellowish brown/ golden brown/ very dark orange brown	Y								●●	
Penning deposits in situ: burnt	Upper (MM1) A	354	354c	15	5	20	2.5	57.5	Sandy loam/ loamy sand. Unsorted. Linked and coated.	PPL: greyish brown/ light brown/ very dark brown; XPL: yellowish brown/ golden brown/ orange brown	Y	Y	Y							
Reworked discard and penning deposits	Upper (MM1) A	New	Na	20	5	25	20	25	Loamy sand. Unsorted. Linked and coated/ Intergrain aggregate.	PPL: light brown; XPL:golden/ yellowish brown.			Y						●●	
Reworked discard and penning deposits	Upper (MM1) B	New	Na	42.5	5	0	12.5	40	Loamy sand. Unsorted. Linked and coated/ Intergrain aggregate.	PPL: light brown; XPL:golden/ yellowish brown.			Y							
Mixed penning and discard material	Upper (MM1) B	New	Nb	20	20	20	7.5	32.5	Loamy sand/ sandy clay loam. Unsorted. Linked and coated and embedded	PPL: light grey/ brown; XPL: golden brown	Y	Y	Y						●●	
Reworked discard and penning deposits	Upper (MM1) B	New	Nc	32.5	27.5	0	7.5	32.5	Sandy clay loam/ loamy sand. Unsorted. Embedded and intergrain aggregate in places.	PPL: light grey brown; XPL: golden brown, light orange brown	Y		Y				●	●	●	
Reworked discard and penning deposits	Lower (MM2) A	354	354a	27.5	5	20	7.5	40	Loamy sand. Unsorted. Linked and coated/ Intergrain aggregate.	PPL: greyish brown; XPL: Yellowish brown/ golden brown			Y						●●	●●
Penning deposits in situ: burnt	Lower (MM2) A	354	354b	15	0	0	65	20	Sand/ loamy sand. Unsorted. Linked and coated.	PPL: greyish brown/ very dark brown; XPL: yellowish brown/ golden brown/ very dark orange brown	Y								●●●	
Penning deposits in situ: burnt	Lower (MM2) A	354	354c	7.5	0	10	2.5	80	Sandy loam/ loamy sand. Unsorted. Linked and coated.	PPL: greyish brown/ light brown/ very dark brown; XPL: yellowish brown/ golden brown/ orange brown	Y	Y	Y						●●	
Penning deposits in situ	Lower (MM2) A	354	354d	17.5	2.5	0	77.5	2.5	Sand. Unsorted. Linked and coated.	PPL: brown/ dark brown; XPL: reddy brown/ very dark reddy brown	Y	Y							●●●●●	
Reworked discard and penning deposits	Lower (MM2) B	354	354e	10	20	20	35	15	Sand/ loamy sand. Unsorted. Linked and coated.	PPL: light brown/ dark brown; XPL: golden brown/ dark reddy brown.								●●●●	●●●●	
Mixed penning and discard material	Lower (MM2) B	354	354f	30	0	10	25	35	Loamy sand. Unsorted. Linked and coated aggregate.	PPL: light brown/ mid brown; XPL: yellowish brown/ mid greyish brown		Y					●●●	●	●●	
Discard deposit	Lower (MM2) B	354	354g	0	0	90	5	5	Loamy sand. Unsorted. Granic	PPL: greyish brown/ light brown/ very dark brown; XPL: yellowish brown/ golden brown/ orange brown			Y						●●	

**Fig. 6:**
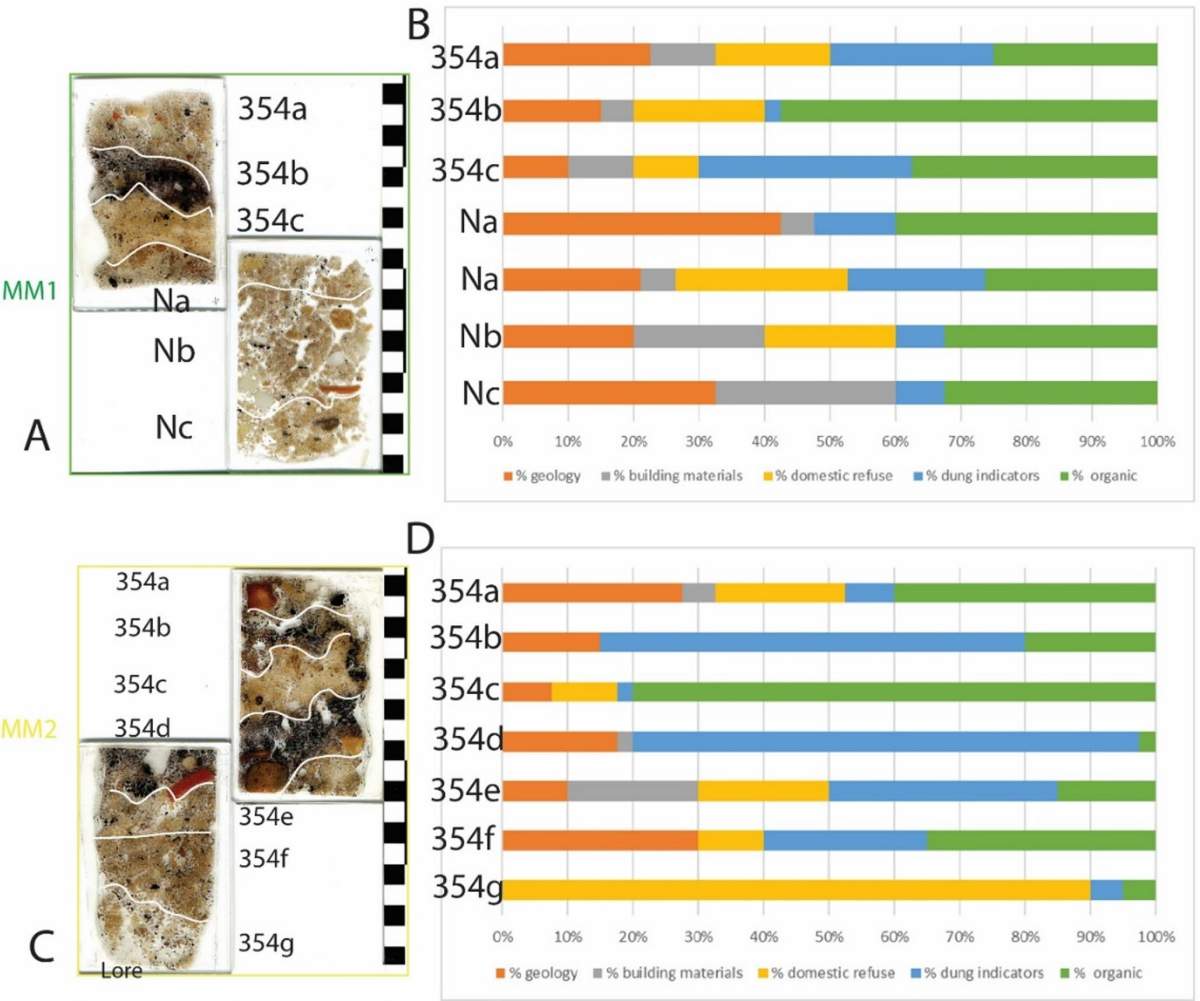
Showing Microstratigraphic Units in MM1 (**A**) and MM2 (**C**) and the abundance of inclusions in the categories of geology, building materials, domestic refuse, dung indicators and organics within each MU (**B** & **D**). MU Na is presented twice as the lowers part contained domestic refuse probably due to mixing with MU Nb below (**B**)

**Fig. 7:**
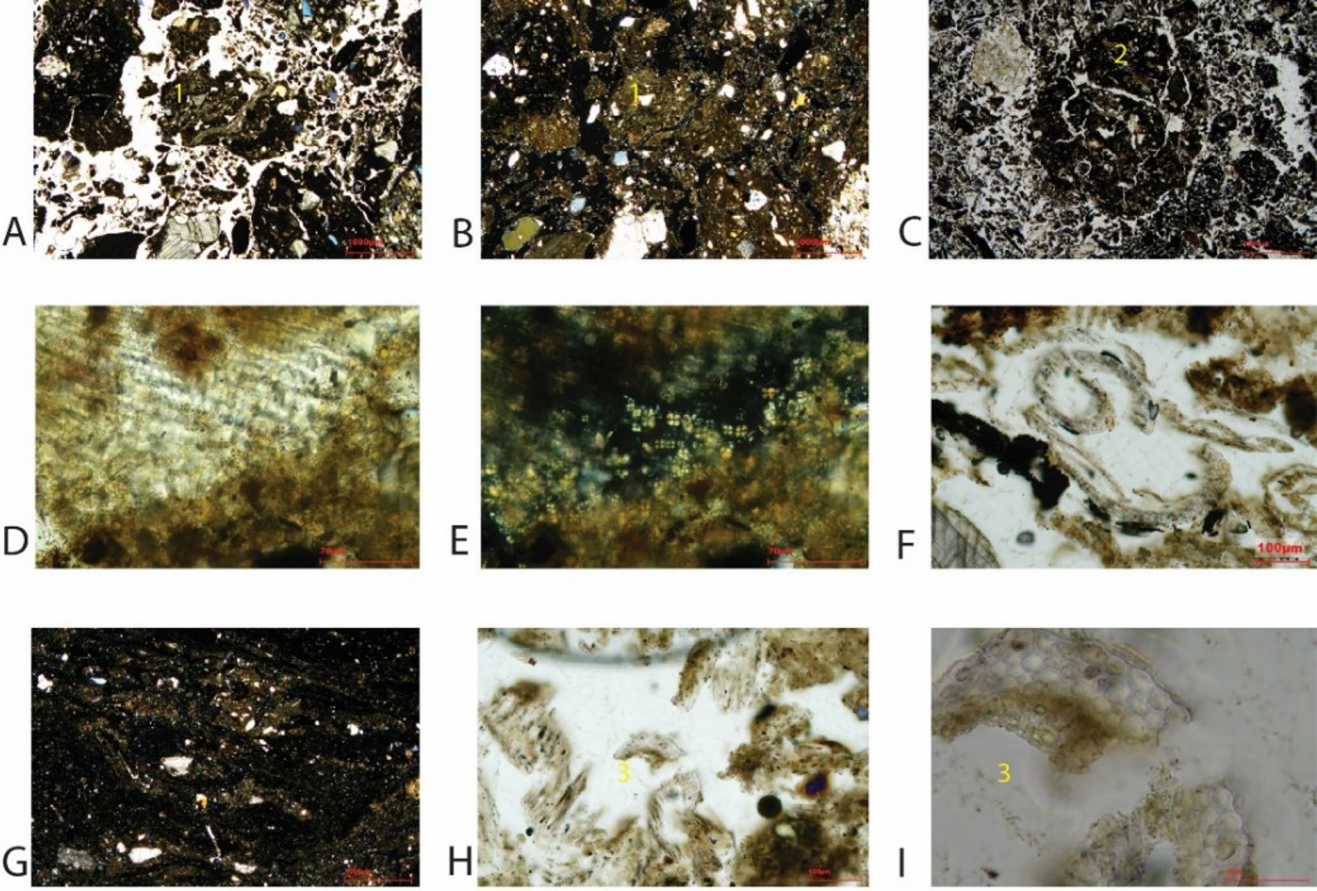
Fragment of herbivore coprolite (Pellet 1), PPL, 25x, MU 354a (**A**), XPL (**B**); Fragment of herbivore coprolite (Pellet 2), PPL, MU 354b (**C**); Contiguous phytoliths and faecal spherulites within Pellet 1, PPL, 25x, MU 354a (**D**), XPL (**E**); Fragment of grass leaf, which has curled due to heating, PPL, MU 354c (**F**); Lenticular platy ped microstructure, XPL, 25x, MU 354c (**G**); Broken contiguous phytolith system, PPL, 200x, MU 354c (**H**), XPL 800x (**I**)

Most MUs are mixed penning and discard deposits (including those that are reworked) and are similar in their composition of inclusions, but with slight variability in the abundances of dung indicators, domestic refuse and fragments of building materials between them (Fig. 6). MU 354c is notable for the higher percentage of organics, and MUs 354b and 354 d are notable for the higher percentages of dung indicators. Dung indicators (Fig. 7A, B, C, E) consist of fragments of herbivore coprolites, which can be burnt, calcareous faecal spherulites and intestinal parasite eggs. Evidence for the presence of organics consists of a range of charred remains (wood, seeds, stems, chaff, unidentifiable and amorphous), vascular bundles, calcitic ashes, melted silica and phytoliths. Large contiguous phytoliths are exceptionally well-preserved; grass leaves were visible in UE 354c, which had curled due to heating (Fig. 7D & F).

The inclusions relating to domestic refuse in the microstratigraphic units comprise fragments of ceramics, bone (burnt and unburnt), fish bone and fish scales, oyster or mussel shell and eggshell (including burnt) and may originate from the piling up then burning of refuse material (Polo-Diaz et al. 2016). MU 354g is an outlier that contains the highest percentage of domestic refuse, and it is classified as solely a discard deposit, which is a secondary deposit (see Schiffer 1987), i.e. representing material that has been removed from its primary place of deposition and redeposited (Matthews 1995; Banerjea et al. 2015a).

MUs 354b, 354c, 354d, Nb and Nc are formed from lenses of organics (dung) and phytoliths (some articulated), which are strongly oriented, aligned to the basal boundary, resulting in a microlaminated bedding structure. This can occur through periodic accumulation (Goldberg and Macphail 2006, pp. 221) when materials have accumulated in their primary place of deposition (see Schiffer 1987), i.e. they are in situ (Matthews 1995; Banerjea et al. 2015a). Evidence for trampling (Rentzel et al. 2017) in the form of broken phytoliths and a lenticular platy ped microstructure (Fig. 7, G, H, I), is observed in MUs Nb, 354c, 354d and 354f.

Bioturbation is evident in several microstratigraphic units, which have been identified in their classification as “mixed” or “reworked” discard and penning deposits (Table 2) to distinguish a slightly greater degree of bioturbation in the latter in the form of well-developed crumb peds, a channelled or chambered microstructure and earthworm calcite biospheriods. Organic staining originating from the decay of organic matter occurs throughout the profile, but it is most abundant towards the base in MUs 354d and 354e.

### Plant macroremains

#### Carpology

**Fig. 8:**
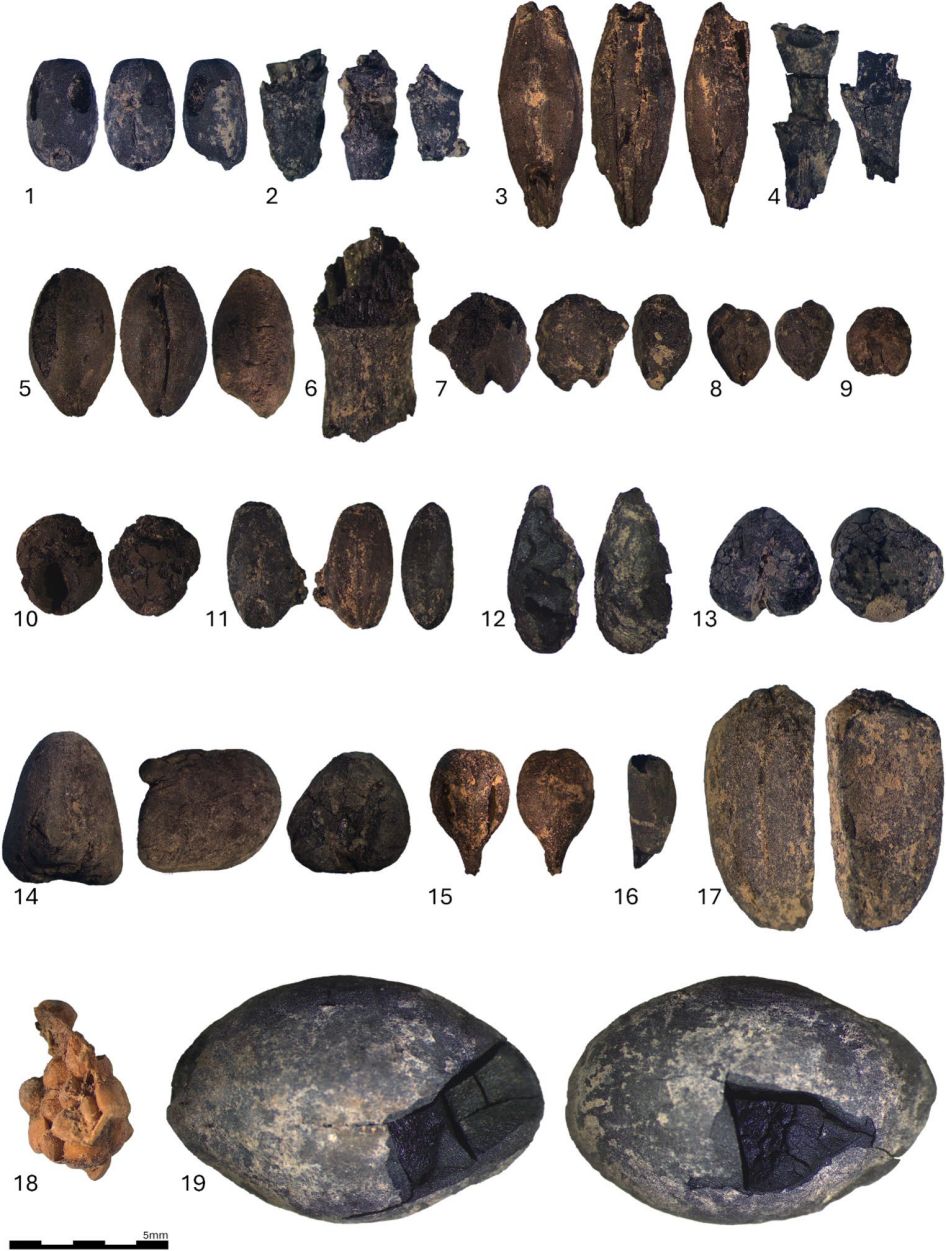
1. Dorsal, ventral and lateral view of *Triticum aestivum/turgidum* 2. Rachis segments of *Triticum aestivum/turgidum* 3. Dorsal, ventral and lateral view of *Hordeum vulgare* 4. Rachis segments of *Hordeum vulgare* 5. Dorsal, ventral and lateral view of *Triticum monococcum* 6. Stem node of Poaceae 7. Dorsal, ventral and lateral view of *Sorghum* sp. 8. Dorsal and ventral view of *Pennisetum glaucum* 9. Dorsal view of *Panicum miliaceum* 10. Dorsal and ventral view of *Sorghum* sp. 11. Dorsal, ventral and lateral view of *Lolium temulentum* 12. Lateral view of *Linum usitatissimum* 13. Frontal and lateral view of *Vicia ervilia* 14. Zenithal, lateral and frontal view of *Vicia faba* var. minor 15. Ventral and dorsal view of *Vitis vinifera* 16. Lateral view of *Punica granatum* 17. *Olea europaea* stone fragment 18. Agglomerated mineralized seeds of *Ficus carica* 19 *Chamaerops humilis* seed

Overall, this preliminary study has revealed the presence in the 6 samples analyzed of 771 carpological remains and 35 taxa (Table 3; Fig. 8). A variety of plant categories is represented: cereals (*Triticum monococcum* L., *T. aestivum/turgidum* L., *Hordeum vulgare* L., *Panicum miliaceum* L., *Pennisetum glaucum* L., *Sorghum* sp., *Avena* sp., cf. *Secale*), cultivated legumes (*Vicia faba* L. var. *minuta*, *V. ervilia* L.), fruit trees and shrubs (*Ficus carica* L., *Olea europaea* L., *Punica granatum* L., *Vitis vinifera* L., *Prunus armeniaca* L.*/dulcis* Mill.), a technical/textile plant (*Linum usitatissimum* L.), as well as wild taxa (*Chamaerops humilis* L., various Amaranthaceae, Apiaceae, Cyperaceae, Poaceae, Malvaceae, Fabaceae), some of which correspond to weeds associated with cereal crops (e.g. *Lolium temulentum*, *Heliotropium europaeum*, *Phalaris* sp.). Cereals are primarily represented by grains, either whole or fragmented, but also by segments of rachis and furcas (naked wheat, hulled barley, einkorn), while fruit trees are predominantly represented by seeds and pits fragments. Although most of the remains are charred, there is also a small, mineralized cluster of fig seeds. As the study is still in its preliminary stage, definitive quantitative comparisons cannot yet be conducted, although einkorn is currently the most abundant cultivated taxon.

**Table 3:** Table carpology: identification and quantification of carpological remains of the corpus (UE = stratigraphic unit; fg. = fragment; X = less than 10 remains; XX = between 10–20 remains; XXX = more than 30 remains)

	Campaign (year)	Unk.	Unk.	2023	2023	2023	2023	
	UE	Seed sample	Seed sample	354.2	354.4	393	394	
	Initial volume (in litres)	Unk.	Unk.	14	18	2,1	1,8	
Taxa	Type of remain							
Charred remains								TOTAL
Cereals, grain and chaff								
*Avena *sp.	grain	−	−	3	−	3	2	8
	grain fg.	−	−	2	−	9	−	11
*Hordeum vulgare*	grain	1	1	4	2	5	1	14
	grain fg.	3	4	3	2	8	3	23
	rachis seg.	−	−	4	1	21	1	27
	lemma base	−	−	−	−	1	−	1
*Hordeum/Triticum*	grain	−	−	−	1	−	−	1
*Panicum miliaceum*	grain	−	−	−	3	1	1	5
*Pennisetum glaucum*	grain	−	−	3	−	1	1	5
cf.* Secale*	grain	−	−	−	−	1	−	1
	grain fg.	−	−	−	−	1	−	1
*Sorghum *sp.	grain	−	−	−	−	−	1	1
*Triticum aestivum/turgidum*	grain	1	4	7	7	−	5	24
	grain fg.	1	2	−	4	−	−	7
	rachis seg.	−	−	−	−	11	−	11
*Triticum monococcum*	grain	70	160	2	−	11	5	248
	grain fg.	45	209	7	−	6	3	270
	furca	−	2	−	−	−	−	2
	furca fg.	−	3	−	−	−	−	3
*Cerealia*	grain fg.	−	−	1	7	−	10	18
Pulses								
Fabaceae (sativa)	seed fg.	−	−	−	−	1	−	1
* Vicia ervilia*	seed	−	−	3	−	−	−	3
cf. *Vicia ervilia*	seed	−	−	−	−	2	1	3
* Vicia faba *var.* minuta*	seed	−	−	−	−	−	1	1
Oily/textile plant								
* Linum usitatissimum*	grain	−	−	−	−	−	3	3
Fruit trees								
* Ficus carica*	seed	−	−	−	1	−	−	1
* Olea europaea*	stone fg.	−	−	5	−	−	−	5
* Prunus armeniaca/dulcis*	stone fg.	−	−	1	−	−	−	1
* Prunus* sp.	stone fg.	−	−		−	−	1	1
* Punica granatum*	seed	−	−	1	−	−	−	1
* Vitis vinifera*	seed	−	−	−	−	5	−	5
	seed fg.	−	−	10	−	4	−	14
	pedicel	−	−	−	−	2	−	2
Wild plants/weeds								
Amaranthaceae	seed	−	−	X	−	−	XX	XX
Apiaceae	seed	−	−	X	−	1	−	X
* Chamaerops humilis*	seed	−	−	1	−	−	−	1
* Cupressus/Tetraclinis*	twig fg.	−	−	2	−	−	−	2
Cyperaceae	seed	−	−	X	−	X	−	X
* Euphorbia* sp.	seed	−	−	X	−	−	−	X
Fabaceae	seed fg.	−	−	−	−	13	−	13
* Heliotropium europaeum*	seed	−	−	X	−	−	−	X
* Lolium temulentum*	seed	−	−	2	−	1	−	3
* Lolium *sp.	seed	−	−	XX	3	XXX	XX	XXX
* Malva *sp.	seed	−	−	X	−	−	−	X
* Medicago *sp.	seed	−	−	X	−	−	1	X
* Phalaris *sp.	seed	−	1	X	−	X	−	X
Poaceae	seed	−	−	XX	−	−	−	XX
	seed fg.	−	−	−	1	−	−	1
	straw node	−	−	4	−	1	−	5
Minralized remains								
Fruit trees								
* Ficus carica*	seed	−	−	−	18	−	−	18
	Total of identified carpological remains	121	386	65	50	109	40	771
	Density (remains/litre)	(no vol.)	(no vol.)	5	3	52	23

#### Anthracology

**Fig. 9:**
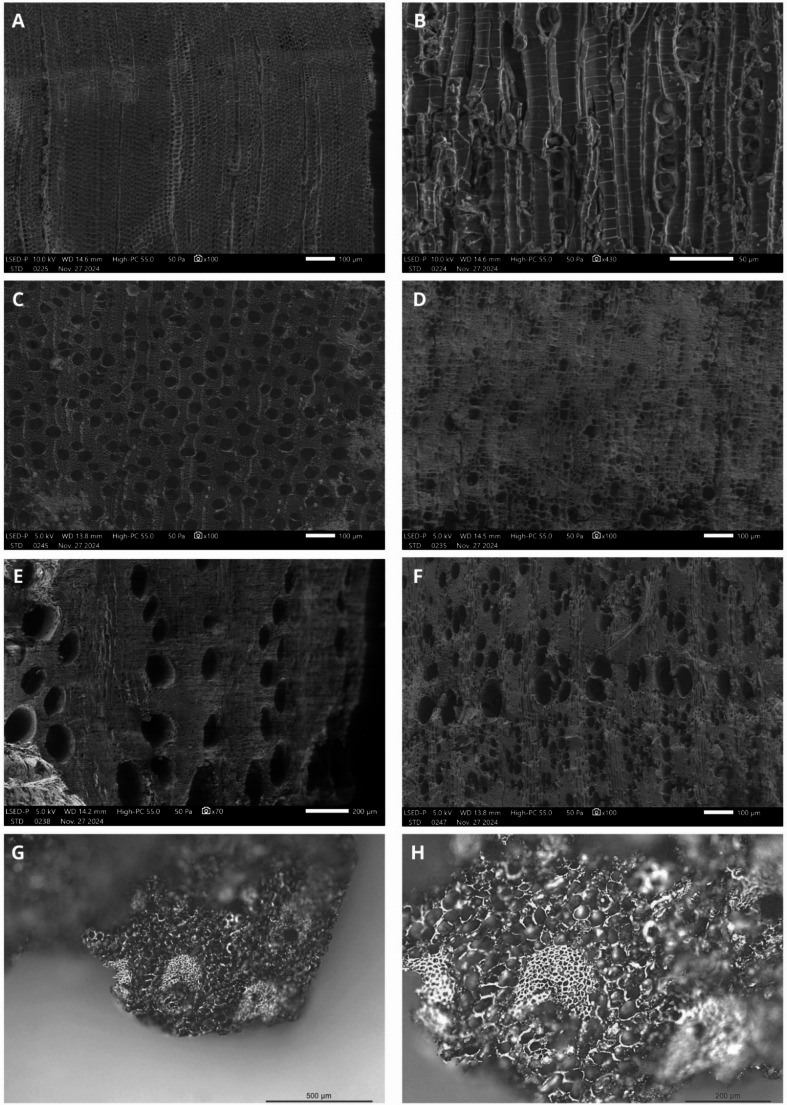
(**A**) Transverse section and (**B**) tangential section of *Taxus baccata*; transverse section of (**C**) Salix sp., (**D**) *Olea europaea*, (**E**) *Quercus suber*, (**F**) *Prunus armeniaca/dulcis/persica*. (**G**) Transverse section of *Chamaerops humilis*, with (**H**) focus on a fibrovascular bundle. Photographs taken (**A**.–**F**.) using the JEOL Hitachi TEM/SEM from the MEA platform, University of Montpellier, and (**G**.-**H**.) using a Nikon camera with an Olympus BH2-UMA microscope

The anthracological analysis focused on layers UE 354.3 and 354.4, yielding 449 charcoal fragments and identifying 18 taxa (Table 4; Fig. 9). The most dominant taxon is tamarisk (*Tamarix*, 18.3%), frequently associated with willow (*Salix*, 14.8%) and poplar (*Populus*, 1.8%) in riparian plant communities. Holm oak (*Quercus coccifera/ilex* L., 15% of the assemblage) represents a typical component of Mediterranean oak woodlands. The assemblage suggests a mixed oak forest, evidenced by the significant proportion of cork oak (*Quercus suber* L., 5.5%) and the presence of yew (*Taxus baccata* L., 0.2%). This woodland composition is accompanied by heliophilous taxa that may form a matorral in more open environments. These include rockrose (Cistaceae, 10.8%), legumes (Fabaceae, 6.6%), pistachio (*Pistacia*, 2%), Atlas cypress (*Tetraclinis articulata* (Vahl) Mast., 1.1%), and Lamiaceae (1.1%), such as rosemary. Other minor taxa include *Phillyrea* and/or *Rhamnus* (0.4%) and strawberry tree (*Arbutus unedo* L., 0.2%) (Rameau et al. 2008). Only two fruit-trees were identified among the fuel remains: olive (*Olea*, 15.6%) and apricot/almond/peach (*Prunus armeniaca/dulcis/persica* L., 1.1%). Additionally, a native palm species, the dwarf palm (*Chamaerops humilis* L.), was identified, with the observed anatomy corresponding to the stipe of the plant (Thomas 2013). The final taxon, acacia (*Acacia*, 2.9%), is not native to the Iberian Peninsula (Blanca and Díaz de la Guardia 1998; Paiva 1999).

**Table 4:** Table anthracology: identification and quantification of the charcoal fragments (L. = Litre)

Structure		"Robber" trench		Total	
Stratigraphic unit		354			
Sample		354.3	354.4		
Volume (L.)		25,2	18		
Context		Open air fumier	Open air fumier		
Chronology		13th century	13th century	N	Occ.
Identifications	*Acacia*	2	11	13	2.9%
	*Arbutus unedo*	1	-	1	0.2%
	Cistaceae	19	28	47	10.4%
	cf. Cistaceae	1	1	2	0.4%
	Fabaceae type *Cytisus/Genista*	16	14	30	6.6%
	Lamiaceae	-	4	4	0.9%
	*Chamaerops humilis*	2	-	2	0.4%
	cf. *Olea europaea*	1	1	2	0.4%
	*Olea europaea*	26	43	69	15.2%
	*Phillyrea/Rhamnus*	2	-	2	0.4%

### Phytolith analysis

#### Phytolith concentrations and preservation

**Fig. 10:**
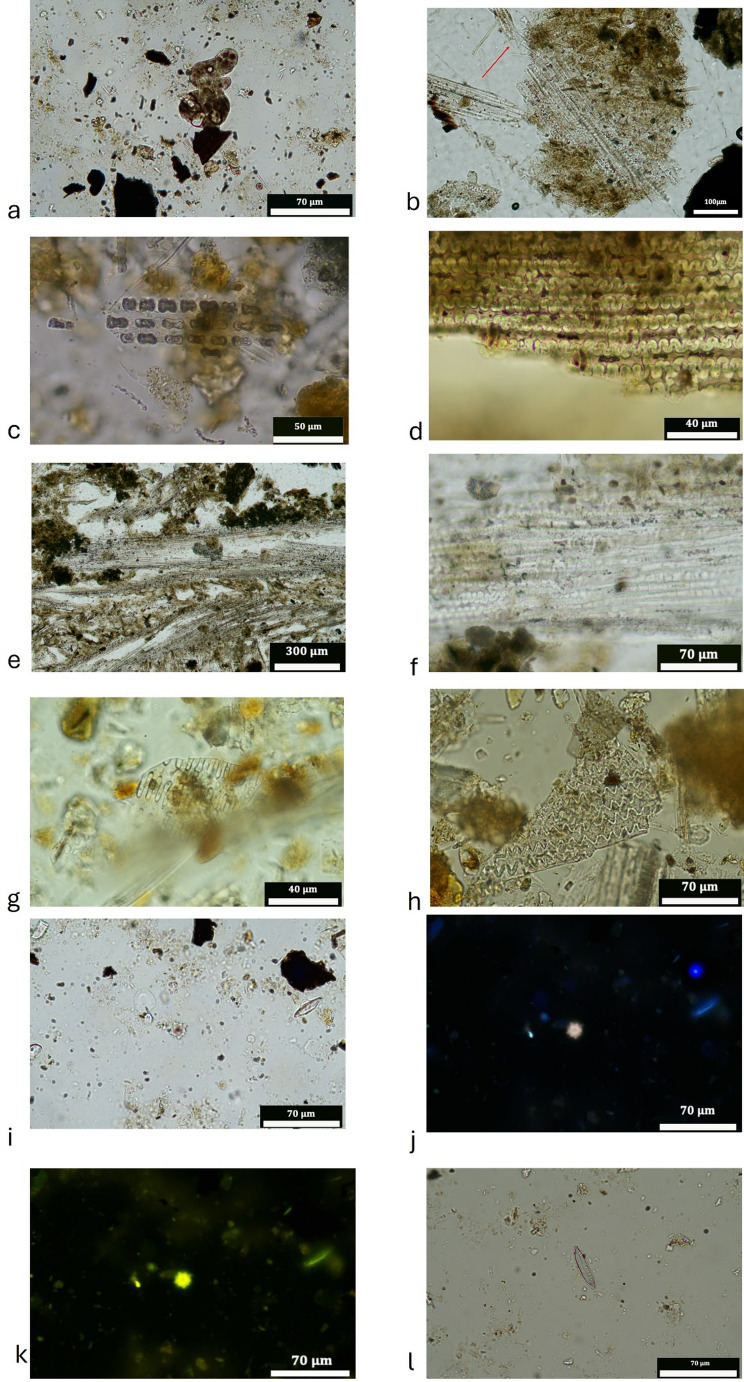
Phytolith microphotographs from soil thin sections and bulk samples (BS) taken at different magnifications: (**a**) Melted silica observed in BS 3 (500x) under PPL; (**b**) Large contiguous system of Elongate dentate from MM2, MU 354b (200x), witnessing the limited postdepositional disturbance (the red arrow points to phytoliths that are no longer showing the same orientation; **c**) Silica skeleton of BILOBATE observed in BS CA2 2024 (500x); **d**) Silica skeleton of Elongate dendritic observed in BS 2, 2019, MU 354c (800x); **e**) Articulated systems of husk phytoliths observed in soil thin section MM2, MU 354c (100x); **f**) Articulated system of Elongate dendritic observed in soil thin section MM2, MU 354c (500x); **g**) Potential millet observed in BS CA2 2019 (800x); h) Silica skeleton from potential threshing sledge cut millet observed in BS CA2 2019 (500x); **i**, **j** and **k**) Palm phytolith Spheroid echinate observed in BS location 3 under PPL(i) UV light (**j**) and blue light (**k**), the auto-fluorescence of the phytolith testifies its heating; **i**) Diatom potentially from genera Nitzschia observed in bulk sample 5 (500x)

The 16 samples analysed yielded phytoliths in high quantities (Sup. Table 4; Table 5). The estimated amount of phytoliths per gram of sediment spans from 3,140,000 in sample 5 (UE 354.2) to 15,080,000 in sample 14 (UE 354.4) (Table 5). The preservation of the phytolith assemblage is exceptional, as the percentage of weathered morphotypes is bracketed between 0 in sample location 9 (UE 354.2) and 4.5% in sample 12 (UE 354.4) (Table 5). Although the percentage of melted phytoliths and vitrified silica (Fig. 10a) is not high when present, auto fluorescent phytoliths appear in relatively high proportions in all the samples, reaching percentages of 55.3% in sample 10 (UE 393) and 56% in sample 3 (UE 354.2) (Table 5).

**Table 5:** List of the 16 samples analysed and their provenience (see Table 1), as well as the results: estimated number of phytoliths per gram of material, number of phytoliths morphologically identified, percentage of weathered phytoliths (chemical alteration), melted phytoliths, vitrified silica and auto-fluorescence phytoliths

UE	Sample location number	#phytoliths per g. of sediment	#phytoliths identified		%weathered morphotypes		%melted phytoliths		%vitrified silica		%auto-fluorescence phytoliths	
354.2	1	7,980,000	362	2.5		0.2		1.8		4.1		
	2	6,510,000	213	1.3		1.3		0.4		34.7		
	3	5,580,000	255	0.3		2.3		-		56		
	4	5,870,000	210	2.3		0.4		-		22		
	5	3,140,000	211	0.4		-		-		34.6		
354.3	6	5,280,000	222	1.2		3.4		0.4		51		
	7	5,080,000	220	0.9		0.4		-		35.4		
	8	6,370,000	242	1.2		-		0.4		45.8		
	9	7,600,000	226	-		0.4		-		30		
393	10	7,580,000	244	1.2		-		-		55.3		
354.4	11	8,440,000	213	0.9		0.4		-		18.7		
	12	11,460,000	203	4.6		0.4		-		30.5		
	13	6,100,000	207	2.3		1		-		10.6		
	14	15,080,000	199	2.4		0.5		-		23.1		
	15	9,510,000	204	2.8		-		-		23		
	16	8,690,000	211	1.8		-		-		21.8

#### Phytolith morphotypes

**Fig. 11:**
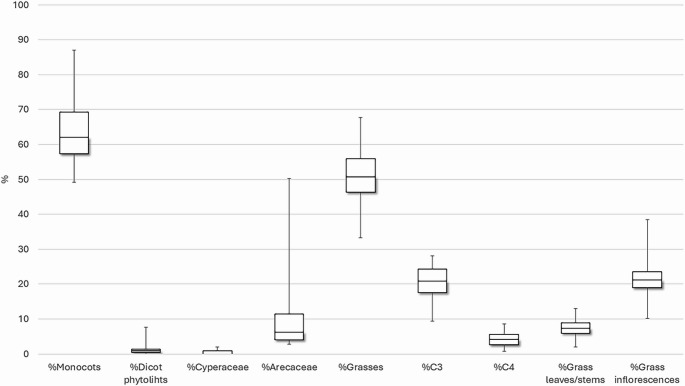
BoxPlot showing the percentage presence of the different phytoliths identified in the phytolith record of Cártama, grouped by their taxonomic and anatomical provenience in plants. The mean values (mid-line), standard error ± (box) and standard deviation (whiskers) are given for the plant types and plant part

Up to 29 morphotypes have been identified in the samples of Cártama. Monocots make up the majority of the phytolith record, while dicot phytoliths are scarce (< 1%). Within the monocot group there are three different families: Poaceae, the most abundant; Arecaceae, which make up 50% of the assemblage in sample 16 (location 3) (UE 354.2); and Cyperaceae which are less abundant (< 1.9%) (Fig. 11).

Poaceae (grasses) have been documented through the presence of the so-called *grass silica short cell phytoliths* (GSSCP) and elongate with different margins. The most common GSSCP are Rondels, produced by the C_3_ Pooideae subfamily (Twiss 1992) to which some of the most important crops, such as barley (*Hordeum vulgare*), wheat (*Triticum* sp.), oat (*Avena sativa* L.), and rye (*Secale cereale* L.) belong to (Zohary et al. 2012). Bilobates were documented to a lesser extent (Fig. 10c). This morphotype is widely produced by Panicoideae grasses, to which millets belong to (Twiss 1992). Bilobate can also be produced by C_3_ plants, as is the case of the Arundinoideae subfamily (reeds), or plants from the Stipeae tribe (Blinnikov 2005; Shakoor et al. 2014).

Phytoliths from grass inflorescences are abundant in the whole phytolith assemblage, but especially in the sample from UE 393 (sample location 10), interpreted as a pit. Particularly Elongate dendritics (Fig. 10d) are traditionally related to the C_3_ Pooideae subfamily crops - especially when their quantity is above 8% (Albert et al. 2008). Hence, their presence is consistent with the documentation of Rondels. Additionally, two different types of elongate traditionally attributed to millets (Lu et al. 2009; Madella et al. 2016) were also documented (Fig. 10g, h). Again, this is consistent with the presence of Bilobate as millets belong to the Panicoideae. Phytoliths produced by the leaves/stems are not as well represented within the phytolith record.

Interestingly, Arecaceae (palm) phytoliths, Spheroid echinate, were documented in all samples (between 2 and 14%). They are especially abundant in sample location 3 (UE 354.2) where they make up the 50% of the plants represented. Moreover, all observed Spheroid echinate are auto-fluorescent in all samples (Fig. 10i, j, k), indicating that these plants were affected by burning processes (Devos et al. 2021; Testé et al. 2025; Vrydaghs et al. 2024).

#### Other silica microremains

Diatoms, single-celled photosynthetic aquatic organisms, were identified in all the samples but location numbers 3, 6 and 16, but in low percentages, between 0.5 and 3.6% in sample location 5 (UE 354.2) (Fig. 10l). We were able to identify only one genera of diatoms: *Nitzschia*. This genus constitutes a very broad group of diatoms that can live in both, fresh and salty water (R. Jahn, Freie Universität Berlin, personal communication). As identification up to species level was not possible, we could not further detail the environmental information provided by these microremains.

### Palynology

**Fig. 12:**
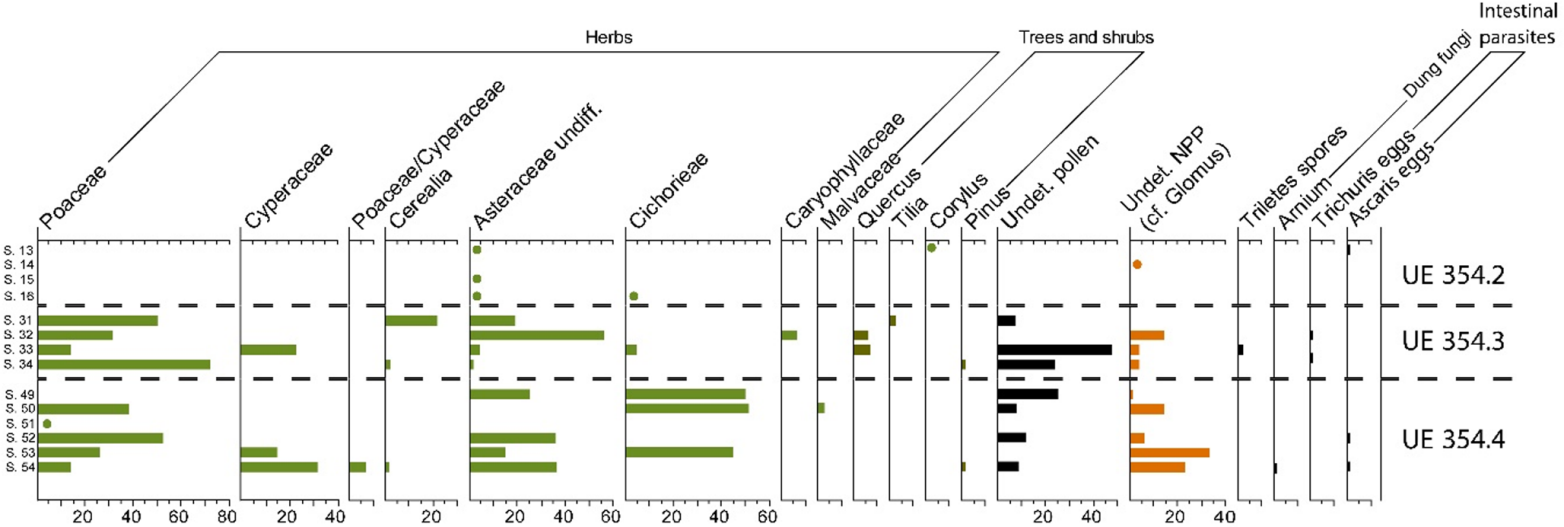
Percentage pollen and non-pollen palynomorph diagram from Cártama, UE 354. The dots mark presence of taxa in levels poor of pollen grains. See Table 1 for corresponding sample location numbers

**Fig. 13:**
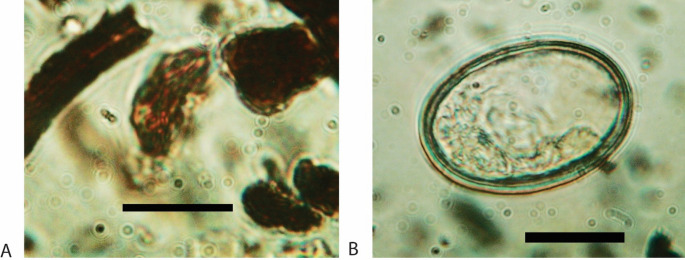
Folded Poaceae pollen, sample 31, (**A**) Decorticated fertilised egg of *Ascaris* sp. (**B**) Scale bar = 20 μm on both images

It was possible to investigate the pollen content of the sequence (Sup. Table 5; Fig. 12), although the grains appeared badly preserved and very often folded and crushed (Fig. 13A), making the identification particularly challenging. The sequence is by far dominated by herbaceous species, in particular wild grasses and sedges, along with herbaceous taxa of the Asteraceae family and its tribe Cichorieae (which include plants like lettuce, dandelions and chicory). Members of the Asteraceae produce very sturdy pollen grains, which tend to be overrepresented in archaeological sites due to their resilience (Florenzano et al. 2012b). Sporadic pollen of arboreal species was also identified, including *Quercus* (morphologically pointing to the Mediterranean evergreen oak, most likely *Q. suber* given the charcoal record), and very rare grains of other species (*Tilia*, *Corylus*, and even *Pinus* pollen).

Eggs of intestinal parasites (*Trichuris* sp., *Ascaris* sp.) were observed in five samples (Fig. 12 & 13B), while spores of dungi fungi were rare (*Arnium*-type). An unidentified palynomorph of uncertain origin resembling small spores of *Glomus* and the morphology of type 179 is relatively abundant throughout the deposit.

### Gas Chromatography/Mass Spectrometry

**Fig. 14:**
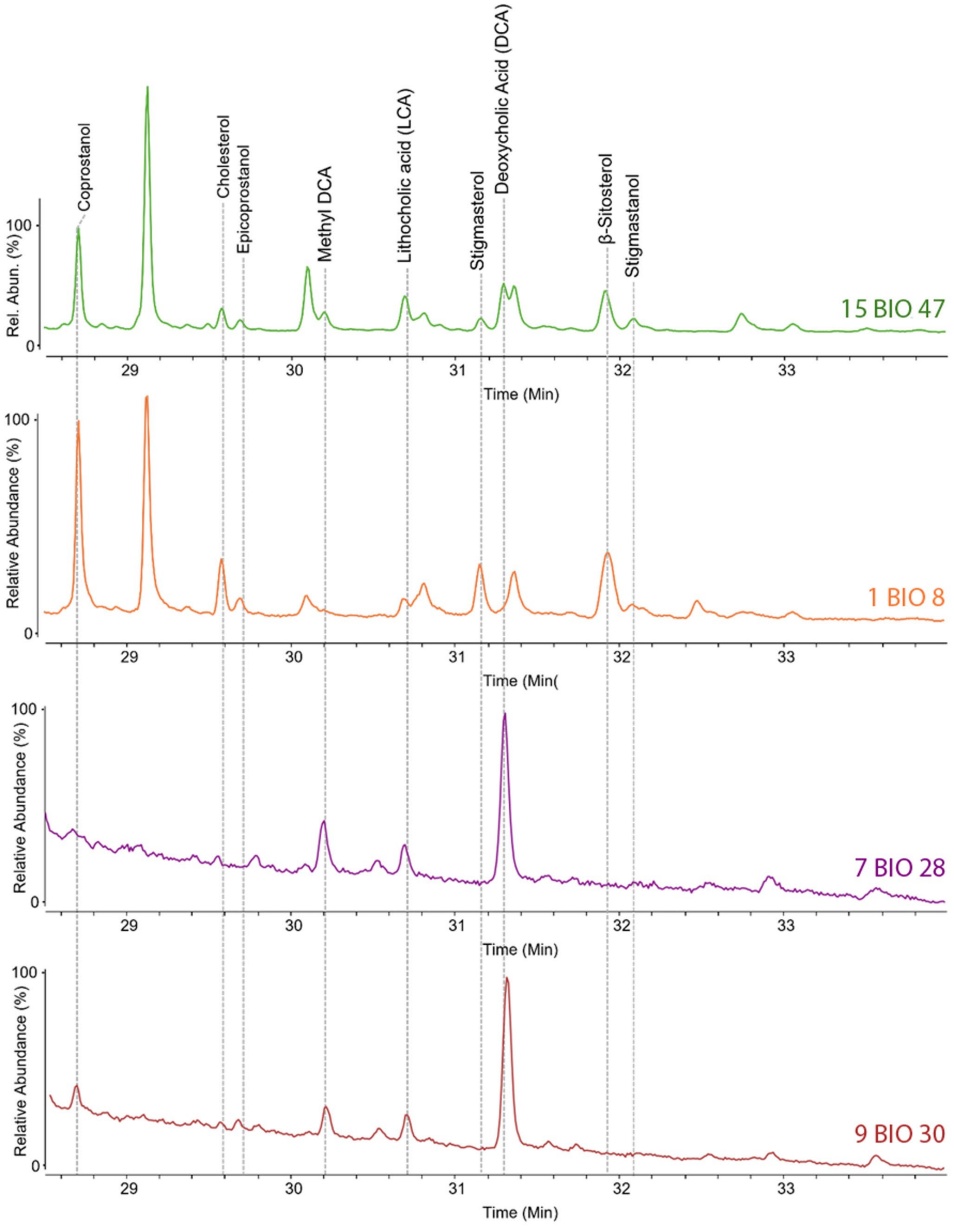
Chromatograms obtained for samples 15 BIO 47, 1 BIO 8, 7 BIO 28 and 9 BIO 30, along with the identified compounds

Although the interpretation of the deposits mostly relies on soil micromorphology, which was applied to the whole sequence, we selected four samples for GC/MS analysis, to better characterise the animal species present on the site. To do so, we focused on the depths where eggs of intestinal parasite were found, assuming that in these levels the signal for the content of compounds from the animal stomachs was particularly intense (1 BIO 8, 15 BIO 47, 7 BIO 28 and 9 BIO 30). The samples were analysed through GC/MS to obtain the chemical profiles of sterols, stanols and bile acids (Table 6; Fig. 14). In particular, bile acids are the most specific markers for faecal input in soil, as they occur exclusively in vertebrate faeces, and their profiles vary among different classes of vertebrates (Bull et al. 2002; Hofmann and Hagey 2008; Reggio et al. 2023).

**Table 6:** Percentage relative abundance based on chromatographic areas for sterols and bile acids in each sample. For each compound are reported the m/z values selected for the generation of its EIC. On the sample ID, the location number appears first followed by the biomarker sample (BIO) number (see Table 1).

	Extracted ions	15 BIO 47	1 BIO 8	9 BIO 30	7 BIO 28
Coprostanol	215, 355, 370	5.0	10.1	0.0	0.0
Cholesterol	329, 368, 458	10.6	34.5	0.0	4.8
Epicoprostanol	215, 355, 370	3.1	5.5	0.0	0.0
Metyl DCA	255, 370, 535	15.4	0.0	24.5	0.0
LCA	215, 257, 430	18.5	0.0	16.4	22.5
Stigmasterol	255, 394, 484	4.2	2.6	0.0	0.0
DCA	255, 345, 428	13.5	0.0	59.1	72.7
Sitosterol	357, 396, 486	25.1	40.8	0.0	0.0
Stigmastanol	383, 473, 488	4.6	6.5	0.0	0.0

### Archaeofaunal remains

The archaeofaunal assemblage recovered from stratigraphic unit UE 354 offers a valuable opportunity to investigate animal husbandry and refuse disposal practices within this periurban Islamic context in southern Al-Andalus. Of the 464 remains analysed, slightly fewer than half (43%, *n* = 199) could be identified taxonomically, with caprines (sheep/goats) forming the core of the assemblage, followed by cattle and a small number of equids, cervids, and pig remains (Table 7). The dominance of caprines, including juvenile individuals and anatomical connections suggesting low post-depositional disturbance, points to localised livestock keeping. However, the presence of butchery marks, signs of marrow extraction, and a notable variety of taxa –including wild species and marine resources– indicates that the deposit also includes refuse from domestic consumption and possibly other economic activities. Particularly striking is the limited but unequivocal presence of pig remains, a species generally absent from Andalusi contexts (García-García 2023); their inclusion here invites further scrutiny regarding their origin and significance. The radiocarbon date of the bone collagen from the pig bone indicates that this is a residual inclusion of prehistoric date, and highlights the caution that must be applied to analysing faunal material from these types of open contexts on urban sites with a long history.

**Table 7:** Absolute and relative frequency of animal species identified (NISP). The category ‘Caprines’ includes all remains identified at the species and subfamily level (*Caprinae*), and so these values (between brackets) are not included in the overall quantification

Class	Taxonomic group	TAXA	NISP	%NISP
Mammals	**Livestock**	Sheep (*Ovis aries*)	11	6
		Goat (*Capra hircus*)	11	6
		Caprines (*Ovis/Capra*)	72	36
		*O/C + OVA + CAH*	*(94)*	*(47)*
		Cattle (*Bos taurus*)	28	14
		Equids (*Equus* sp.)	5	3
		Pig (*Sus* sp.)	3	2
		*Subtotal livestock*	*(130)*	*(65)*
	**Wild**	Rabbit (*Oryctolagus cuniculus*)	4	2
		Lagomorphs	2	1
		Red deer (*Cervus elaphus)*	4	2
	**Commensal**	Dog (*Canis familiaris)*	1	1
Birds	**Domestic**	Chicken *(Gallus domesticus*)	25	13
	**Wild**	Pigeon (*Columba* sp.)	1	1
		Partridge (*Alectoris rufa)*	1	1
Fish (unid.)		nID	14	7
Malacofauna (unid.)		nID	17	9
Total			**199**	**100**

### Summary of integrated results

The integrated results for UE 354.2, 354.3 and 354.4 (Table 8) permit discussion below of the evidence for burning, the in situ nature of the evidence for the corral, the diet and health of the livestock, other uses of vegetal resources and seasonality. The micro- and bio-archaeological data are examined in the context of the deposit classification and formation processes, as observed using soil micromorphological analysis, which identified and classified eight additional microstratigraphic units. These MUs were only identified in the profile, and they did not have any additional samples collected from them.

**Table 8:** Summary of integrated results from stratigraphic units (UE) 354.2, 354.3, and 354.4

UE	MU	Micromorphology deposit type	Faecal lipid markers	Plant macroremains	Silica microfossils	Pollen and npps
354.2	354a	Reworked discard and penning deposits	Sterols and stanols	Wide variety of charred plant species in the carpological remains including cereal grains, notably *Pennisetum glaucum*, segments of rachis, pulses, fruit seeds and stones, and wild plants/weeds.	Palm phytoliths, which are affected by burning, made up 50% of the assemblage (sample location 3). Phytoliths from grass inflorescences, particularly ELONGATE DENDRITICS. Silica skeletons of millet. Diatoms.	Intestinal parasite eggs (*Ascaris*)
354.3	354b	Penning deposits in situ: burnt	Bile acids: lithocholic (LCA) and deoxycholic acids (DCA)	The main taxa in the anthracological assemblage (in number of fragments) are *Tamarix*, *Quercus coccifera/ilex*,* Quercus suber*,* Salix*,* Olea europaea*. Two fragments of *Chamaerops humulis* (including a stipe) were also found.	Phytoliths from grass inflorescences, particularly ELONGATE DENDRITICS. Phytoliths show autofluorescence from burning.	Pollen assemblage is dominated by herbaceous species, in particular wild grasses and sedges, along with herbaceous taxa of the Asteraceae family, with sporadic tree pollen, mainly *Quercus*. *Cerealia* type pollen occurs in sample 31. Intestinal parasite eggs (*Trichuris*).
354.4	354c	Penning deposits in situ: burnt	Bile acids and coprostanol, epicoprostanol	A cluster of 18 mineralised fig seeds were identified in the carpology assemblage. Similar taxa in the anthracology assemblage to SU 354.3, but notably 11 fragments of *Acacia*, which is not native to the Iberian Peninsula.	Phytoliths from grass inflorescences, particularly ELONGATE DENDRITICS. Potential millets. Phytoliths show autofluorescence from burning.	Dung fungi spores (*Arnium*- type). Intestinal parasite eggs (*Ascaris*).

Two pits were identified cutting into the *fumier* sequence and the carpology and phytolith assemblages within their fills are distinct from the layers of the *fumier,* and indicate that these pits predominantly contained grain. The carpology assemblages show a greater variety and abundance of cereals, charred grains and chaff, and fewer fruit remains than in the penning deposits comprising UE 354 and include *Panicum milaceum*,* Pennisetum glaucum*,* Secale*,* and Sorghum* sp. Phytoliths from grass inflorescences traditionally related to crops are abundant in the whole phytolith assemblage, but especially in the sample from UE 393. No micromorphological, GC/MS or palynological analyses were conducted on the fills from the two pits (UE 393 and UE 394). The anthracological assemblages were not examined as part of the preliminary study. The pits may have functioned as shallow feeding pits in the form of makeshift containers for grain for livestock within the corral.

The presence of livestock is evidenced by fragments of herbivore dung and a suite of dung indicators in thin section, faecal lipid markers, and coprophilous fungal spores and eggs of intestinal parasites in extracted microfossils. The preliminary zooarchaeological evidence shows the dominance of caprines, a prevalence of juvenile remains, and, taphonomic aspects such as the anatomical articulation of bones, and traces of trampling. Evidence for trampling is also observed in the soil micromorphological data, specifically in MUs Nb, 354c, 354 d and 354f (Table 2).

The presence of pig has been discussed in relation to the identification of coprostanol and the limited size of *Trichuris* eggs allow us to restrict the hosts only to swine or humans (Beer 1976; Arobba et al. 2018; Nielsen et al. 2021). The presence of pig remains within the archaeofaunal assemblage does not permit any further corroboration for the presence of pigs within the corral. The radiometric dating of pig collagen produced a prehistoric date showing this to be residual material.

## Discussion

### Evidence of burning

Soil micromorphological analysis shows a range of inclusions that have been transformed by heating: fragments of herbivore coprolites, bone, fish bone, plant remains (wood and non woody), melted phytoliths and vitrified silica. Although the percentage of melted phytoliths and vitrified silica is not high when present in the phytolith extractions, auto fluorescent phytoliths appear in relatively high proportions in all the samples, reaching percentages of 56% in sample location 3 (UE 354.2) In thin-section, some of the inclusions are charred and partially burnt. It is possible that some of these inclusions, such as charred wood and bone from domestic fires and activities, have been transformed pre-deposition, particularly in those deposits that are classified as mixed discard and penning deposits. Several documentary sources from writers of Andalusi agronomy, such as Ibn Bassal (1955: 55–60), Ibn Wāfid (Millás, 1943: 281–332; 1948: 345–430, 3005) and Ibn al-‘Awwān (1864, 2003; I, 123–139), discuss the preparation of manure for the use on fields. These include preparation methods using additives such as household waste, hearth ash, sweepings and urine, timeframes whereby manure is left to mature, and the use of hollows or trenches where the manure is left.

The charred plant macro-remains in the anthracology assemblage, including both seeds and charcoal fragments, exhibit a remarkable diversity of taxa originating from various environments and operational sequences. This raises questions about the origins of these remains, the locations and conditions under which they were burned, and the processes that led to their mixing within the studied assemblages. The presence of ash- and charcoal-rich layers within the manure suggests the incorporation of waste from hearth cleanings or combustion contexts, potentially as a means of sanitizing the area by absorbing odours and moisture (Milek 2012). It remains challenging to distinguish between materials that were burned prior to deposition and those potentially combusted in situ; however, several microstratigraphic units show features indicating that these deposits have formed *in situ.*

### A corral in situ

The presence of dung, livestock alimentation, in situ depositional processes evidenced by lenses of organics (dung) and phytoliths (some articulated), which are strongly oriented, aligned to the basal boundary, resulting in a microlaminated bedding structure, and indications of trampling make the case for this feature, UE 354, being a small corral, where livestock sheltered taking advantage of the hollowness of a “robber trench”, occupying an area of approximately 4.00 m x 3.40 m.

Soil micromorphological analysis shows that dung indicators (fragments of herbivore coprolites, which can be burnt, calcareous faecal spherulites and intestinal parasite eggs) occur in all microstratigraphic units in the profile (Figs. 6 and 7). A wide range of residues and micro-particles have been identified that indicate that both herbivore and possibly omnivores (pig or human) faecal waste are also present, which, when also considering the presence of pig in the faunal assemblage, is particularly interesting in an Andalusi context; although it is approached with caution here as radiometric dating of pig collagen produced a prehistoric date. Analytical chemistry also contributed to the reconstruction of the farming activities and of the animal assemblage. Although without the quantitative analysis of sterols and stanol and bile acids it is difficult to clearly assert the class of vertebrates to which belong the faecal material, some consideration can be done.

In sample 1 BIO 8 only sterols and stanols are detected, while in sample 7 BIO 28, 9 BIO 30 and 15 BIO 47 also bile acids can be identified. The detection of bile acids such as lithocholic (LCA) and deoxycholic acids (DCA), together with its derivate methyl deoxycholate (Methyl-DCA), suggests the presence of faecal input. However, even if bile acids are not detected in sample 1 BIO 8, also in this sample a faecal input could be assumed, since specific stanols generated in the gut of higher animals, coprostanol and epicoprostanol, are detected.

Focusing on sample 15 BIO 47, further information can be obtained from the chromatographic profile. For that aim, the percentage composition based on chromatographic areas of each sample is reported in Table 6. The table also reported the m/z values used to generate the specific EICs of each analyte. The detection of sitosterol and other phytosterols along with bile acids point to the presence of herbivores at the site. In addition, since the chromatographic abundance of DCA together with metyl-DCA is higher than that of LCA, it may be hypothesised that these herbivore animals were ruminant (Prost et al. 2017; Reggio et al. 2023). The trend of an higher percentage of DCA compared to LCA could be observed in all the three sample where bile acids were detected This fact, together whit the presence in sample 1 BIO 8 and 15 BIO 47 of coprostanol and epicoprostanol, a major sterol in human and swine, may indicate the presence also of omnivores, even if others characteristic compounds, such as cholic acid and chenodeoxycholic acid, are not detected (Prost et al. 2017; Reggio et al. 2023).

Eggs of intestinal parasites (*Trichuris* and *Ascaris* sp.) are relatively ubiquitous throughout the sequence (Figs. 12 and 13B). The finding of *Ascaris* eggs among the untreated sediment samples confirms that this taxon is particularly susceptible to laboratory preparations and more prone to destruction (Banerjea et al. 2021). The limited size of *Trichuris* eggs allow us to restrict the hosts only to swine or humans (Beer 1976; Arobba et al. 2018; Nielsen et al. 2021). This is consistent with the presence of coprostanol and epicoprostanol identified by GC/MS analyses. Pigs have been reported at Islamic period sites, although in low frequencies compared to Christian sites (Alexander et al. 2019; Grau-Sologestoa 2017; García-García 2023; Morales et al. 2011;). On the other hand, we cannot rule out the possibility of human faecal inputs being discarded on the site, therefore both possibilities remain plausible, but unconfirmed by the archaeofaunal assemblage. As for the presence of diatoms in penning deposits, including *fumier* sequences, they can derive from the drinking water source after being ingested by the animals (Brochier et al. 1992; Macphail et al. 1997; Banerjea et al. 2015b).

The carpology sample from UE 354.4 contains a cluster of mineralized fig seeds (Fig. 18:8), preserved through a process that requires two specific conditions. The first is irregular water presence in the sediment, such as alternating phases of immersion and drying, variations in moisture within the structure, or proximity to a fluctuating water table (Green 1979). The second condition is the presence of organic remains (e.g., faecal matter, plant material, bones, cartilage) or calcareous and calcium phosphate-rich elements, including metals or ceramics (Green 1979). When these conditions are met, dissolved mineral substances penetrate plant tissues through water contact and, via precipitation, gradually replace the organic matter. This highly selective preservation mode depends on seed characteristics and tissue composition. Not all seeds are capable of mineralizing; fruit-bearing plants producing robust seeds (e.g., stones, pips) are particularly well-suited to this form of preservation. In this case, mineralization likely resulted from the presence of animal faecal matter.

In situ formation processes were observed in the soil micromorphological analysis whereby MUs 354b, 354c, 354 d, Nb and Nc are formed from dung and organic materials have accumulated in their primary place of deposition. This means that residues, micro- and macro-fossils recovered from bulk samples within UE354.3 and UE354.4 have micro-contextual support (Banerjea et al. 2019) to state that there is a strong possibility that they are in their primary place of deposition. Given the high percentage of auto-fluorescent phytoliths, it is also probable that a high proportion of organic materials have been burnt in their primary place of deposition in these stratigraphic units, which can be seen by the leaf of a grass that curled due to heating in MU345c/UE354.4.

**Fig. 15:**
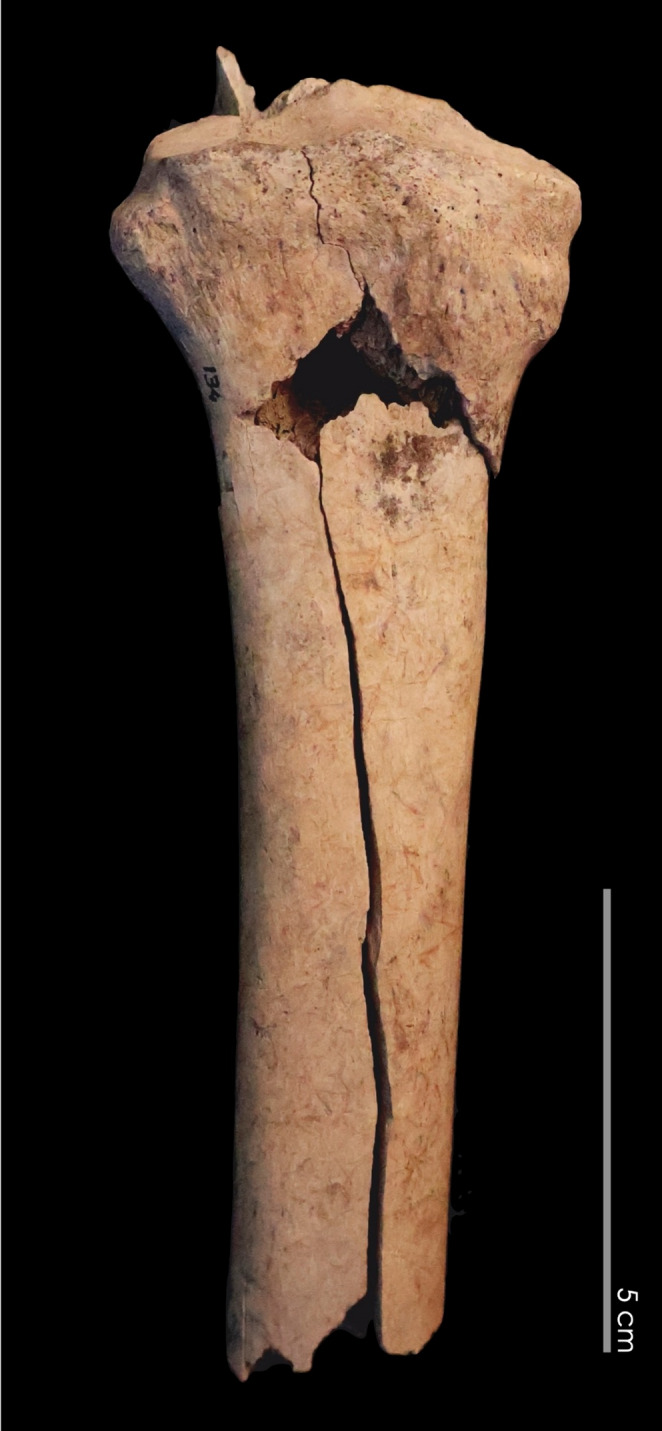
Radius of Equus showing evidence of ancient fracturing, typically attributed to trampling or other post-depositional attritional processes

The very scarce presence of dung fungal remains, despite abundant dung layers, may be an indication that animals were relatively continuously kept in the area, causing surface disturbance with trampling activity (Morandi 2025). Evidence for trampling was also observed during soil micromorphological analysis in the form of a lenticular platey ped microstructure and broken phytoliths (Fig. 7G). Importantly, the zooarchaeological evidence –especially the prevalence of juvenile remains, the anatomical articulation of bones, traces of trampling (Fig. 15), and the overall composition of the assemblage– strongly aligns with the stratigraphic and micromorphological interpretation of the feature as a *fumier* sequence and further indicates a low level of post-depositional disturbance. This convergence of taphonomic, taxonomic, and contextual indicators supports the identification of UE 354 as a structured accumulation of livestock-related waste, likely associated with an outdoor corral or yard. Although the sample size does not yet allow for detailed demographic or biometric conclusions, the evidence already points to a complex pattern of animal use that encompassed not only meat production and on-site husbandry but also the consumption of hunted and fished species.

To summarise, the analyses show evidence for the use of the hollow as an corral, and evidence for additives, as described in the documentary sources by Ibn Bassal (1955: 55–60), Ibn Wāfid (Millás, 1943: 281–332; 1948: 345–430, 3005) and Ibn al-‘Awwān (1864, 2003; I, 123–139), to prepare fertiliser containing manure for use on agricultural fields. It may be that this space was used as part of a cycle of stabling, dung burning and the maturing of manure containing additives for the use on fields.

### Diet

The carpological assemblage contains a broad range of cultivated plants, including cereals, legumes, flax, and fruit-bearing taxa. However, it remains unclear whether these remains are associated with animal feed or originated from domestic combustion structures, later introduced into the penning deposits through the deposition of ash or charcoal. The presence of pearl millet (*Pennisetum glaucum*) and sorghum (*Sorghum bicolor*) is particularly noteworthy (Fig. 8: 7–10), as these are medieval Muslim introductions that remain rare in the Iberian Peninsula (Pérez-Jordà et al. 2024). Millets or potential millets were identified in the phytolith record from UE 354.2 and UE354.4, as well as phytoliths from grass inflorescences that are traditionally related to crop (Fig. 10b, d, e, f). Further morphometric analyses will be performed on phytoliths in soil thin sections in order to detail botanical attribution of these crops (Vrydaghs et al. 2016). Sample 2 from 2019 (UE 354.2) produced an articulated silica skeleton of millet (Fig. 10h) that had been cut by a threshing sledge (Anderson 2003). These are abundant in the whole phytolith assemblage, but especially in the sample from UE 393 (pit). Additionally, bitter vetch (*Vicia ervilia*) is identified in the carpology, a species primarily associated with animal feed and rare in medieval contexts in the peninsula (Peña-Chocarro et al. 2019). It is of note that UE 354.4 has been identified using micromorphology as in situ penning deposits, so carpological and phytolith remains in this sample have a very strong probability of relating to animal feed. It is also of note that sorghum was only recovered from pit fill (UE 394) not the *fumier* contexts. Both the carpological and phytolith assemblages from the pits (UE 393 and 394) are different in composition to the penning deposits comprising UE 354 and indicate that the pits may have functioned as temporary feeding pits containing grain for livestock.

### Health indicators

If the coprostanol and epicoprostanol identified by GC/MS analyses and the *Trichuris* eggs derive from human stools, we may hypothesise that domestic refuse was discarded on the area, and that mild symptoms associated with trichuriasis and ascarisis affected the local population (Morandi 2018; Mitchell 2024); otherwise, it indicates infection of the local livestock population, which here comprises predominantly caprines including juvenile individuals and anatomical connections suggesting low post-depositional disturbance, points to localised livestock keeping. Cattle and a small number of equids, and cervids also occur in the archaeofaunal assemblage.

### Use of vegetal resources

In palaeoecology, palynology is primarily used to reconstruct past landscapes; however, as attested in other archaeopalynology studies, this assemblage cannot be considered representative of any existing past habitat due to issues of taphonomy and differential preservation in the archaeological layers, but can reveal information about activities on a site (e.g. Banerjea et al. 2020; Expósito and Burjachs 2016). The abundance of herbaceous taxa, along with the occurrence of sporadic clusters of grass pollen, suggests that whole flowers were layered down with intact plant material (e.g. grass bundles) to create a vegetal bedding (Macphail et al. 1997; Mazzucco et al. 2022; Hunt et al. 2023). This is consistent with the hypothesis of a fodder deposit in relation to stabled animals, which following burning tend to form a typical sequence normally referred to as *fumier* in pastoral archaeology (Angelucci et al. 2009) Articulated phytoliths in thin sections (Fig. 10e & f) demonstrate that plants were laid down either as bedding or surface cover, as also observed at Arene Candide (Macphail et al. 1997).

Although the pollen record mostly reflects the fodder composition and the animal diet (herbs), it also captures the existence of mixed oak forests characterized by cork oak in association with lime trees around the site. Sporadic pollen of pine was probably transported over larger distances (a few tens of km) and higher elevations. The anthracological analysis also highlights the presence of local taxa, including riparian vegetation, mixed oak forests, and matorral. However, certain taxa raise questions. For instance, yew (*Taxus baccata*) (Fig. 9A & B) is typically restricted to montane or submontane zones but may occur at lower altitudes under specific conditions. Its presence in the assemblage prompts inquiries regarding its origin and use, whether as timber or fuel. In sheepfolds, yew charcoal is reported to be abundant specifically in caprine dung layers and becomes scarcer in strata associated with the rise of cattle breeding or non-pastoral activities, and its use has been reported as insecticide, as per the antibacterial properties of its wood (Ruiz-Alonso et al. 2017; Delhon et al. 2024 and references therein). *Acacia*, an exotic taxon, is another intriguing presence. It was likely introduced into the Iberian Peninsula during the medieval period and utilized for multiple purposes, including wood production, gum, tannin, dyes, perfumes, flowers, and ornamental planting (Paiva 1999).

Arecaceae phytoliths were prevalent in the assemblage and the dwarf palm, *Chamaerops humilis*, occurs in the macrobotanical assemblage (charred seed, Fig. 8: 19, and stipe, Fig. 9G & H). The case of Arecaceae phytoliths (Spheroid echinate) (Fig. 10i, j, k) in UE 345.2 (sample location 3, Fig. 5A) is particularly interesting. This UE is pale/white and is the latest of the sequence and is deposited above the last blackened unit, thus this is the layer that closes the stabling episode. Hence, it can be interpreted that palms were the main fuel used as tinder to start the fire (Polo-Díaz et al. 2016; Alonso-Eguiluz et al. 2017, 2024); although the penning deposits have been reworked with some discard material. Unfortunately, it is not possible, with the information available up to date, to make a more accurate taxonomical and anatomical attribution of this phytolith morphotype (Albert et al. 2009; Vrydaghs et al. 2001).

### Seasonality

The plant remains in the studied micro- and macrobotanical assemblages do not permit a clear reconstruction of the seasonality associated with the formation of the penning deposits. The taxa exhibit diverse growth cycles:

- Cereals: Winter and spring/summer cereals are all harvested in summer but may be stored for short, medium, or long-term use.
- Fruit trees: Taxa such as grapevine, olive, fig, and pomegranate can be consumed at various times of the year. Additionally, many fruits can be preserved through drying. Carpological analysis cannot determine whether the remains originated from fresh or dried fruits.
- Wood: Woody remains can be harvested year-round. Notably, the presence of fruit tree wood suggests potential pruning waste, although such materials could also be stored for later use.

Traditionally, inflorescence phytoliths have been used to assess seasonality, as they are related to the blooming season. However, the high presence of grass inflorescence phytoliths might also indicate that animals were being fed, at least partially, with crops secondary by-products, making difficult to stablish the season of use, since they can be stored for future uses. Some research suggest that the livestock is stabled during the spring-summer, while others propose longer periods of occupation (Alonso-Eguiluz et al. 2017, 2024; Cano-Cano et al. 2022; Martín et al. 2022). Hence additional proxies must be analysed in the future to better understand the seasonality of this open-air *fumier* deposit.

## Conclusion

Multidisciplinary analyses of late medieval deposits from suburban Cártama have, for the first time, confidently identified open-air *fumier* deposits. This demonstrates that Andalusi livestock husbandry practices included periodically burning animal dung within corrals and pens, which is a long-standing practice in the Mediterranean region also observed in prehistoric transhumance. Like prehistoric rock-shelter *fumier* deposits, domestic refuse was also discarded into the corral, albeit more prevalently in this medieval urban setting, maybe for the purpose of enhancing the fertiliser. It is possible that these deposits were removed periodically and used on agricultural fields for soil amelioration. Our findings shed new light on a fundamental aspect of Andalusi society and economy, which is absent in the written sources relating to pastoral practices. The identification of a broad range of plant remains in the deposits demonstrate the integral relationship between plants and animals, essential for an incremental reappraisal of the medieval “Green Revolution”. In particular, the presence of pearl millet and sorghum provides an important temporal and spatial marker for mapping the diachronic distribution of these key agrarian introductions in the Western Mediterranean.

## Supplementary Information

Below is the link to the electronic supplementary material.

Supplementary Material 1

Supplementary Material 2

Supplementary Material 3

## Data Availability

No datasets were generated or analysed during the current study.
